# Genetic variation in *P-*element dysgenic sterility is associated with double-strand break repair and alternative splicing of TE transcripts

**DOI:** 10.1371/journal.pgen.1010080

**Published:** 2022-12-07

**Authors:** Jyoti Lama, Satyam Srivastav, Sadia Tasnim, Donald Hubbard, Savana Hadjipanteli, Brittny R. Smith, Stuart J. Macdonald, Llewellyn Green, Erin S. Kelleher

**Affiliations:** 1 Department of Biology and Biochemistry, University of Houston, Houston, Texas, United States of America; 2 Department of Molecular Biology and Genetics, Cornell University, Ithaca, New York, United States of America; 3 Department of Molecular Biosciences, University of Kansas, Lawrence, Kansas, United States of America; Fred Hutchinson Cancer Research Center, UNITED STATES

## Abstract

The germline mobilization of transposable elements (TEs) by small RNA mediated silencing pathways is conserved across eukaryotes and critical for ensuring the integrity of gamete genomes. However, genomes are recurrently invaded by novel TEs through horizontal transfer. These invading TEs are not targeted by host small RNAs, and their unregulated activity can cause DNA damage in germline cells and ultimately lead to sterility. Here we use hybrid dysgenesis—a sterility syndrome of *Drosophila* caused by transposition of invading *P-*element DNA transposons—to uncover host genetic variants that modulate dysgenic sterility. Using a panel of highly recombinant inbred lines of *Drosophila melanogaste*r, we identified two linked quantitative trait loci (QTL) that determine the severity of dysgenic sterility in young and old females, respectively. We show that ovaries of fertile genotypes exhibit increased expression of splicing factors that suppress the production of transposase encoding transcripts, which likely reduces the transposition rate and associated DNA damage. We also show that fertile alleles are associated with decreased sensitivity to double-stranded breaks and enhanced DNA repair, explaining their ability to withstand high germline transposition rates. Together, our work reveals a diversity of mechanisms whereby host genotype modulates the cost of an invading TE, and points to genetic variants that were likely beneficial during the *P-*element invasion.

## Author summary

Transposable elements (TEs) are mobile genetic parasites that spread through host species’ genomes by making additional copies of themselves in developing gametes. Transposition requires the breakage of DNA to introduce a new TE copy, which burdens the host cell to repair the damage or tolerate the mutation. To avoid these fitness costs, eukaryotic hosts suppress transposition in developing gametes through small-RNA mediated silencing. However, what happens when a new TE that is not recognized by existing small-RNAs invades the genome? We examined genetic variation in the degree to which *Drosophila melanogaster* oogenesis is disrupted by the transposition of a newly invading TE family. We show that while transposition results in gamete loss in some genotypes, other genotypes maintain fertility. Furthermore, we show that increased fertility likely reflects both decreased permissivity of transposition, as well as increased tolerance of DNA damage. Our observations provide a window into how host genetic variation impacts the consequences of invading TEs.

## Introduction

Transposable elements (TE) are mobile DNA sequences that spread through host genomes by replicating in germline cells. Although individual TE insertions are sometimes beneficial, genomic TEs are foremost genetic parasites reviewed in [1]. Unrestricted transposition not only produces deleterious mutations, but also double-stranded breaks (DSBs) that lead to genotoxic stress in developing gametes. The mechanisms by which hosts enact silencing of resident TEs through the heritable production of regulatory small RNAs is extensively studied and broadly conserved [2,3]. However, host genomes are frequently invaded by new TE families, against which they lack small RNA-mediated “immunity” [4–7]. In the context of such novel TEs, genetic variation in the host’s ability to produce gametes could be a critical determinant of fitness. The presence and mechanisms of such host variation remain largely unstudied.

*P-*element DNA transposons, which invaded natural populations of *Drosophila melanogaster* around 1950, provide a unique opportunity to uncover host genetic variation in transposition-dependent sterility [8–10]. Strains isolated from natural populations prior to this invasion, referred to as M strains, do not contain genomic *P*-elements, and do not produce maternally-transmitted piRNAs that control their expression and transposition. When females from M strains are mated to males bearing genomic *P*-elements (P-strains), they produce dysgenic offspring that do not negatively regulate *P-*elements in germline cells [11]. A range of fertility effects result from unregulated *P*-element transposition, including the complete loss of germline cells and sterility [12]. However, naive M genotypes differ in their propensity to produce dysgenic progeny suggesting genetic variation in dysgenic sterility [8,10,13,14].

One potential source of variation in *P-*element dysgenic sterility arises from the response of germline cells to DSBs arising from transposition. In dysgenic females, primordial germ cells (PGCs) are lost beginning in the second instar larval stage, most likely due to unrepaired DSBs [15–17]. Furthermore, mutations in DNA damage response and repair proteins are known to enhance dysgenic germ cell loss [18,19]. Therefore, it is predicted that genotypes that enact more efficient DSB repair should be more tolerant of *P-*element transposition, and maintain germline cells. A related response is the production of *de novo* piRNAs, which is triggered by the activation of the DNA damage response protein checkpoint kinase 2 (CHK2) in the adult female germline [20,21]. These *de novo* piRNAs transcriptionally silence *P-*elements in a process analogous to maternally transmitted silencing, and restore fertility as dysgenic females age [20,21]. If *de novo* piRNA production activates more readily in some genotypes than others, it could lead to fertility differences in dysgenic crosses.

Another potential source of variation in dysgenic sterility lies with host co-factors of transposition, including host proteins that regulate the transcription and splicing of transposase-encoding RNA, or the activity of *P-*element transposase enzyme. In particular, differences in splicing cofactors between germline and somatic cells ensure *P-*element transposase-encoding transcripts are only produced in germline cells [22]. However, individual germlines could differ in the production of these cofactors. Beyond transposase production, *P-*elements insert preferentially near origins of replication, a strategy that may facilitate their spread through the genome by ensuring they are replicated multiple times in a given S-phase [23]. Differences in the timing or composition of this machinery could therefore drive differences in transposition rates, and ultimately downstream germline loss.

We recently isolated natural variation in dysgenic sterility through QTL mapping, using a panel of highly recombinant inbred lines derived from M strains (*Drosophila* Synthetic Population Resource, DSPR, Population A RILs, [24]). We mapped a major effect QTL surrounding the gene *bruno*, a female germline differentiation factor [14]. Here we present results from a second QTL mapping study in an independent panel of DSPR RILs (Population B, [24]). We describe two QTL that determine differences in dysgenic sterility, one in young females only, and one in aged females only. Focusing on young dysgenic females, we further interrogated mechanisms underlying fertility differences by contrasting RNA and small RNA expression, radiation sensitivity, and *P*-element expression and splicing between fertile and sterile genotypes. Our results suggest that natural variation in dysgenic sterility arises through differences in both germline DNA repair and *P-*transposase mRNA splicing, revealing considerable complexity in host factors that modulate the fitness costs associated with transposition.

## Results

### QTL mapping

**Fig 1:**
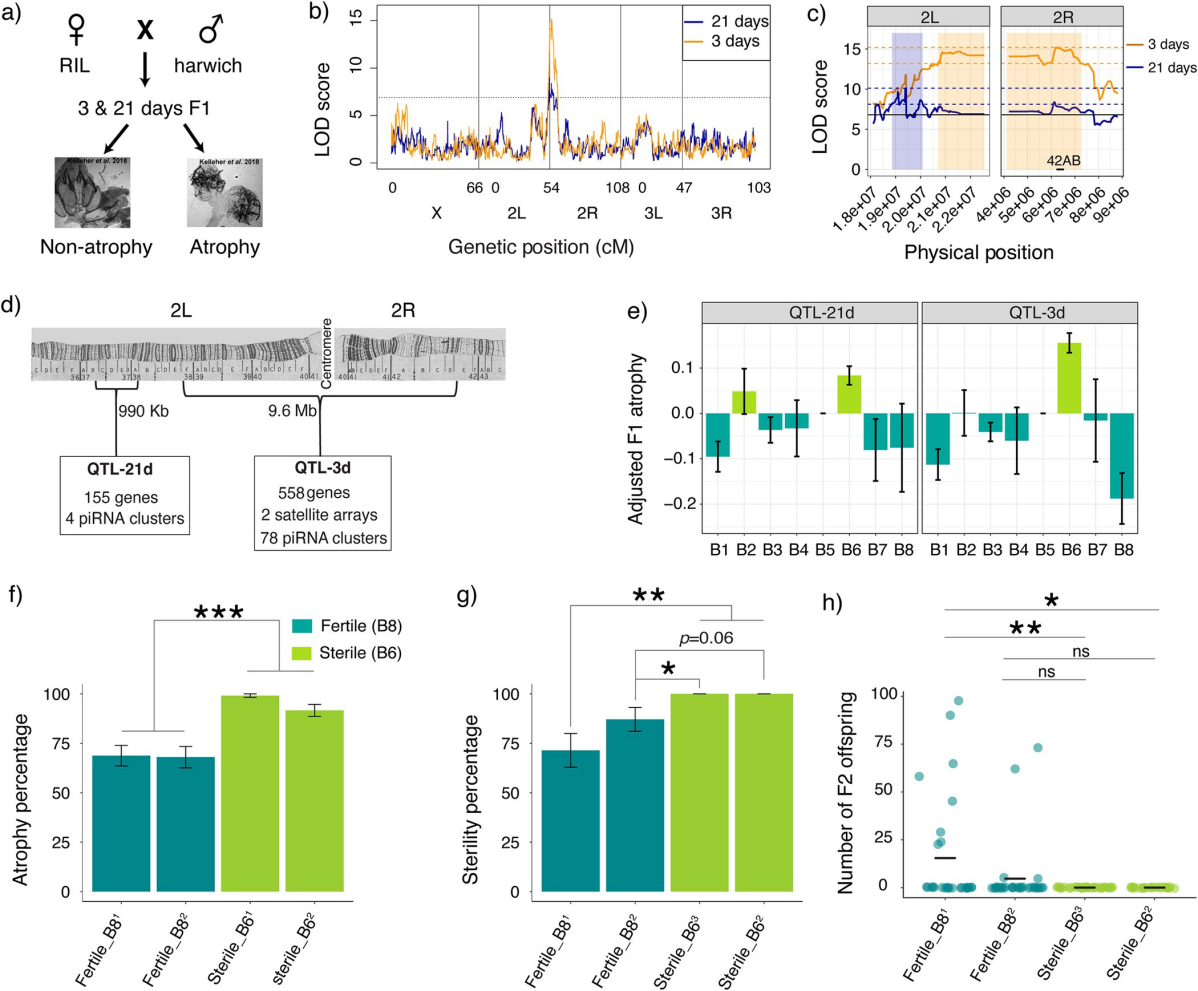
QTL mapping of variation in *P*-element induced ovarian atrophy. **a)** Crossing scheme to phenotype the variation in F1 ovarian atrophy among RIL offspring. Representative images of atrophied and non-atrophied ovaries are from Kelleher *et al. [14]* **b)** The log of odds (LOD) plot for QTL mapping of ovarian atrophy using 3 day-old (gold) and 21 day-old (blue) F1 females. The dotted line is the LOD threshold and x-axis represents the chromosomal positions. **c)** Zoomed-in figure of QTL mapping from 3 days (gold) and 21 days (blue). The colored boxes show the genomic interval that likely contains the causative genetic variant of each QTL, based on a **Δ**2LOD drop from the peak position [26]. The pairs of dotted lines indicate the peak **Δ**2LOD scores that determines the interval. The solid horizontal line is the LOD significance threshold based on 1,000 permutations of the phenotype data. **d)** Cytological map depicting the interval of the two QTL peaks [27,28]. **e)** Residual F1 atrophy (y-axis) associated with each of the eight founder alleles (x-axis) at the QTL peaks after accounting for random effects. All the QTL peaks show 2 phenotypic classes: sterile (light green) and fertile (dark green). **(f-g)** Percentage of **(f)** ovarian atrophy and **(g)** sterility among dysgenic female offspring from crosses between Harwich males and isogenic females carrying sterile (B6) and fertile (B8) alleles. Proportions were compared between samples using χ2 tests of independence. **h)** Number of F2 offspring produced by individual dysgenic F1 females from crosses between Harwich males and isogenic fertile and sterile females. The horizontal line indicates the mean, which was compared between samples using permutation tests. Superscripts1,2 and 3 in (**f-h**) denote isogenic lines that were independently generated, these sometimes differ between experiments because sterile_B61 became contaminated and was replaced with sterile_B63. Error bars in **e, f** and **g** represent the standard error. The data used to generate plot in panels **b**,**c**, and **e** are provided in **S3 and S4 Tables** and that used for plot in panels **f**, **g and h** are provided in **S17** and **S18 Tables** respectively. * denotes P < 0.05, ** denotes P < 0.01, *** denotes P < 0.001.

The DSPR RILs are all *P-*element free M-strains, which were derived from founders isolated from natural populations before the *P*-element invasion [24]. We therefore screened for alleles that influence dysgenic sterility among the panel B RIL genomes by crossing RIL females to males from the reference P-strain Harwich, and examining the morphology of F1 ovaries (**Fig 1A)**. Atrophied ovaries are indicative of germline loss resulting from *P-*element activity [14,25]. Since dysgenic sterility changes across development [15], and some females exhibit age-dependent recovery from *P-*element hybrid dysgenesis through the production of *de novo* piRNAs [20], we phenotyped F1 females at two developmental time points: 3 days and 21 days post-eclosion.

Similar to our observations with the Population A RILs [14], we found continuous variation in the frequency of ovarian atrophy among dysgenic offspring of different RIL mothers, indicating genetic variation in dysgenic sterility that is unrelated to maternally deposited piRNAs (**S1 and S2 Tables**). Based on a combined linear model of F1 atrophy among 3 and 21 day-old females, we estimated the broad-sense heritability of dysgenic ovarian atrophy in our experiment to be ~42.5%. The effect of age on the proportion of F1 atrophy was significant but minimal (*χ*2 = 7.03, df = 1, p-value = 0.008) with 21 day-old females showing only 0.7% increase in atrophy as compared to 3 day-old females. This suggests that age-dependent recovery from dysgenic ovarian atrophy through the production of *de novo* piRNAs is not common among the genotypes we sampled.

To identify the genomic regions associated with genetic variation in dysgenic ovarian atrophy, we performed QTL mapping using the published RIL genotypes [24]. We found a large QTL peak near the 2nd chromosome centromere in both 3 and 21 day-old F1 females **(Fig 1B and Tables 1, S3, and S4).** However, the genomic intervals of the two QTL are non-overlapping **(Fig 1C and Table 1)**. The major QTL in 21 day-old females (hereafter, QTL-21d) resides in the euchromatic region and is quite small (990 kb) compared to the major QTL in 3 day-old females (hereafter QTL-3d), which spans the centromere and pericentromeric regions (9.6 Mb, **Fig 1D**). Therefore, there are likely at least two polymorphisms that influence tolerance near the 2nd chromosome centromere, one of which has a larger effect in young 3-day old females, with the other having a larger effect in 21 day-old females.

**Table 1:** QTL positions in 3 and 21-day old females. The peak position, **Δ**2LOD drop confidence interval (2LOD CI), and the Bayesian Credible Interval (BCI) in dm6 [29] are provided for each analysis. The data used to identify the LOD peaks and intervals for 3 and 21-day old females can be found in **S3** and **S4 Tables**, respectively.

Analysis	LOD Score	Peak Position	2LOD CI	BCI	% variation
3-day	15.2	2R:6,192,495	2L:20,710,000-2R:7,272,495	2L:20,820,000-2R:6,942,495	11.13
21-day	10.13	2L:19,420,000	2L:19,170,000- 20,080,000	2L:19,010,000- 20,000,000	9.78

We further evaluated the effect of the two linked QTL through haplotype analysis. We modeled residual F1 ovarian atrophy as a function of QTL haplotype for the 3 day and 21 day peaks, thereby disentangling synergistic (e.g. sterile 3d, sterile 21d) from opposing (e.g. sterile 3d, fertile 21d) allelic combinations (**S4 Fig**). We observed that the 3 day-old QTL allele is solely-determinant of ovarian atrophy in the 3 day-old offspring. However, in 21 day-old offspring only the genotypes containing fertile alleles at both QTL show decreased atrophy. This suggests that QTL-3d may determine germ cell maintenance in the larval, pupal and early adult stages, but QTL-21d may be additionally required to maintain germline cells in aging females. The presence of two QTL is further supported by the phenotypic classes we detected among founder alleles (B1-B8) for each of the QTL peaks (**Fig 1E**). For QTL-21d, both B2 and B6 founder alleles are associated with greatly increased dysgenic ovarian atrophy. By contrast for QTL-3d, only the B6 founder allele is associated with increased ovarian atrophy.

We next sought to determine whether reduced ovarian atrophy corresponds to restored fertility, or merely allows for the production of inviable gametes. To this end, we generated isogenic lines that carry either high-atrophy (B6) or low-atrophy (B8) alleles at both QTL loci in an otherwise identical genetic background through 6 generations of backcrossing to a marker stock (**S5 Fig**). Consistent with our QTL mapping, B8 alleles display less F1 ovarian atrophy (24–31%) than B6 strains when crossed with Harwich males (**Fig 1F and S17 Table**). Furthermore, while B6 dysgenic females produced no offspring, 13–29% of dysgenic B8 females were fertile and produced offspring (**Fig 1G and S18 Table**). For one B8 stock, offspring counts were significantly higher when compared to B6 (**Fig 1H).** In light of these observations, we refer to the low-atrophy and high-atrophy alleles hereafter as “fertile” and “sterile”. The fertility rescue conferred by these alleles would be highly beneficial in populations where dysgenic crosses are common.

### Sterile and fertile alleles differ in chromatin regulation

**Fig 2:**
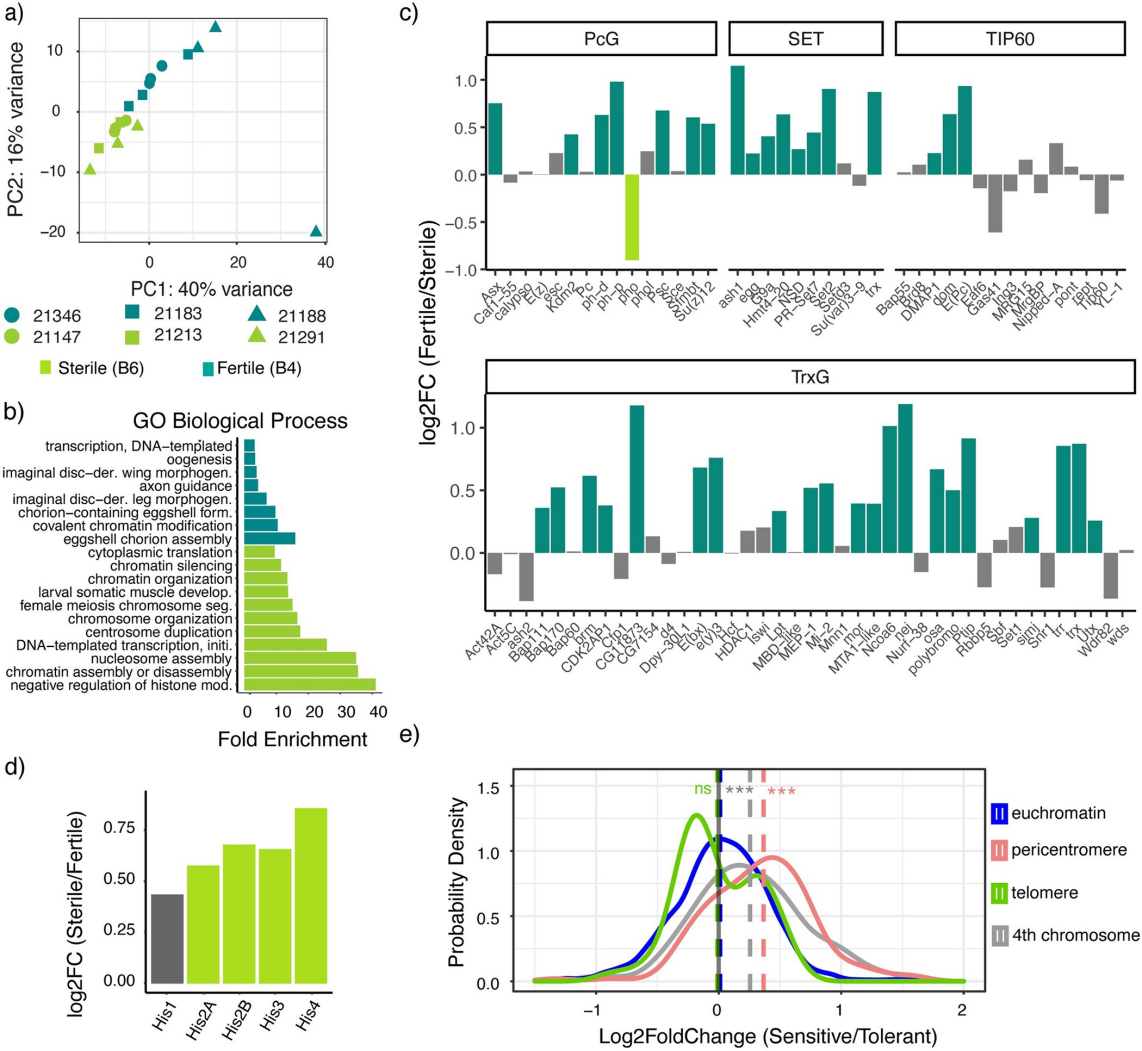
Fertility is associated with increased chromatin modification, whereas sterility is associated with increased expression of replication-dependent histones. **a**) PCA analysis of gene expression data for pairs of sterile (B6) and fertile (B4) RILs, that carry founder B6 and B4 haplotypes across the QTL window. Members of the same RIL pair with otherwise similar genetic backgrounds are represented by the same shape. **b)** GO terms enriched among genes upregulated in fertile and sterile genotypes. **c)** Log2 fold differences in expression for chromatin modifiers between sterile and fertile genotypes. **d)** Log2 fold increase in RD histone expression in sterile genotypes. **e)**. Probability density plot of log2 fold change values for all euchromatic (blue), pericentromeric (red), telomeric (green) genes and 4th chromosome (gray) between strains carrying sterile and fertile. The mean of each distribution is represented by a dotted line, and is compared between distributions with a two-sample *t*-test. The x-axis boundaries were confined from (-1.5 to 2) for a better visualization. The pericentromere-euchromatin boundaries were drawn from [29,45] and subtelomeric-euchromatin boundary coordinates from [46–48]. The data represented in panel **a** is provided in **S14 Table** and plot in panels **c, d,** and **e** in **S5 Table**). *** denotes P < 0.001.

Both the QTL regions contain large numbers of protein coding and non-coding RNA genes, piRNA clusters, and repeats, which could influence dysgenic sterility (**Fig 1D**). To better understand the differences between fertile and sterile genotypes, we compared their gene expression profiles in the ovaries of young 3–5 day-old females by stranded total RNA-seq. To avoid the confounding effects of germline loss under dysgenic conditions, we focused on RIL females rather than their dysgenic offspring. To account for potential background effects, we examined three pairs of RILs that carried either a sterile (B6) or fertile (B4) QTL haplotype across the QTL region (dm6 2L:19,010,000-2R:7,272,495) in otherwise similar genetic backgrounds (shared 44–47% of founder alleles outside the QTL). Please note that these lines differ from the B6 and B8 isogenic stocks we utilize in **Fig 1** and later in the manuscript, which are more closely matched for genetic background. Principal component analysis (PCA) of read counts reveals two independent axes that resolve sterile and fertile gene expression profiles, which together account for 40% and 16% of variation (**Fig 2A and S14 Table**). One biological replicate of RIL 21188 (fertile) was an outlier, which we excluded from our downstream analysis of differentially expressed genes.

We found a total of 530 genes differentially expressed between sterile and fertile genotypes (Benjamini-Hochberg adjusted *p*-value < = 0.05, fold-change > 1.5; **S5 Table**). The most significantly enriched gene ontology (GO) term among genes upregulated in fertile ovaries is chorion assembly (Bonferroni corrected *P* value <0.01, **Fig 2B and S7 Table**). This suggests a larger number of late-stage oocytes in fertile ovaries, as other genes that are upregulated in late oogenesis (stages 12–14) show a similar increase in expression (**S6 Fig,** [30]). Because atrophy results from the loss of larval PGCs and pre-meiotic adult cysts (GSCs), larger numbers of late-stage oocytes are likely unrelated to dysgenic sterility [15–17,19].

The second most significant GO term upregulated in the ovaries of fertile genotypes is covalent chromatin modification (**Fig 2B**). Strikingly, we discovered fertile ovaries exhibit a systematic upregulation of multiple chromatin modification complexes with key roles in oogenesis, including polycomb group, trithorax group, and the TIP60 complex, as well as many individual SET domain lysine methyltransferases (**Fig 2C**) [31–33]. The TIP60 complex in particular is involved in cell cycle progression and differentiation in pre-cystoblasts [32]: daughter cells of germline stem cells in which *P-*elements transpose [19,21]. Interestingly, the TIP60 complex is also involved in DSB repair [34], which could promote dysgenic germ cell survival. Similarly, polycomb-dependent gene silencing initiates in nurse cells concurrently with meiosis I: a window in which germ cell cysts with large numbers of DSBs undergo apoptosis [35].

Genes upregulated in the sterile genotypes are enriched for functions in chromatin assembly and transcription, cell division, and translation. However, a careful inspection of genes underlying these enriched terms reveals that, with the exception of translation, they are primarily explained by the increased expression of replication-dependent (RD) histone gene copies (**Fig 2D**). While these expression increases are modest (<2 fold), they are likely an underestimate of the true degree of histone upregulation. Histone gene expression increases dramatically in late-stage oocytes (beyond stage 10 [36]), which appear to be reduced in the ovaries of sterile genotypes (**S6 Fig**). Overexpression of RD histones is associated with increased sensitivity to DNA damage [37–41], and excess histones are reported to compete with DNA repair proteins for binding to damage sites [38]. Thus, while TIP60 activity might increase DSB repair in fertile ovaries promoting germ cell survival, histones might decrease repair in sterile ovaries increasing germ cell death.

Differences in ovarian chromatin modification between fertile and sterile genotypes may further be connected to histone regulation. Replication-dependent histones occur in a tandemly duplicated gene cluster that exhibits coordinated and dosage-compensated regulation in a specialized nuclear compartment known as the histone locus body (HLB, [42]). In particular, negative regulation of histone expression relies on multiple heterochromatin factors [41,43]. Consistent with reduced heterochromatin formation in the ovaries of sterile genotypes, they show higher expression of pericentromeric genes (two-sample t-test, *t141* = -9.32, *p*-value = 2.3x 10–16), as well as genes on the heterochromatic 4th chromosome (two-sample t-test, *t53* = -4.56, *p*-value = 3.0x10-5, **Fig 2E**). The sterile B6 haplotype also exhibits increased expression of pericentromeric genes in a previously published microarray dataset from head tissue ([44] **S1 Fig**).

### Sterile genotypes exhibit silenced piRNA loci, but no systematic TE dysregulation

In addition to gene expression, differences in the regulation of resident TEs could modulate the degree of dysgenic sterility. The *D*. *melanogaster* genome harbors >100 resident TE families [49,50], many of which are transpositionally active and show variable transposition rates in wild-type strains [51–53]. If sterile alleles establish weaker regulation of some resident TEs, their transposition could add to DNA damage resulting from *P-*element transposition, thereby promoting germ cell loss. Resident TEs are regulated by piRNAs, and two features of our data suggest differences in piRNA biogenesis between sterile and fertile alleles. First, QTL-3d contains numerous piRNA clusters, including the major ovarian piRNA cluster *42AB*, which could differ in the regulation or resident TEs between sterile and fertile alleles (**Fig 1D)**. Second, differences in chromatin regulation between sterile and fertile alleles could impact piRNA cluster expression (**Fig 2E and 2F**), which is dependent upon the heterochromatic histone modification, histone 3 lysine 9 trimethylation (H3K9me3) [54,55].

**Fig 3:**
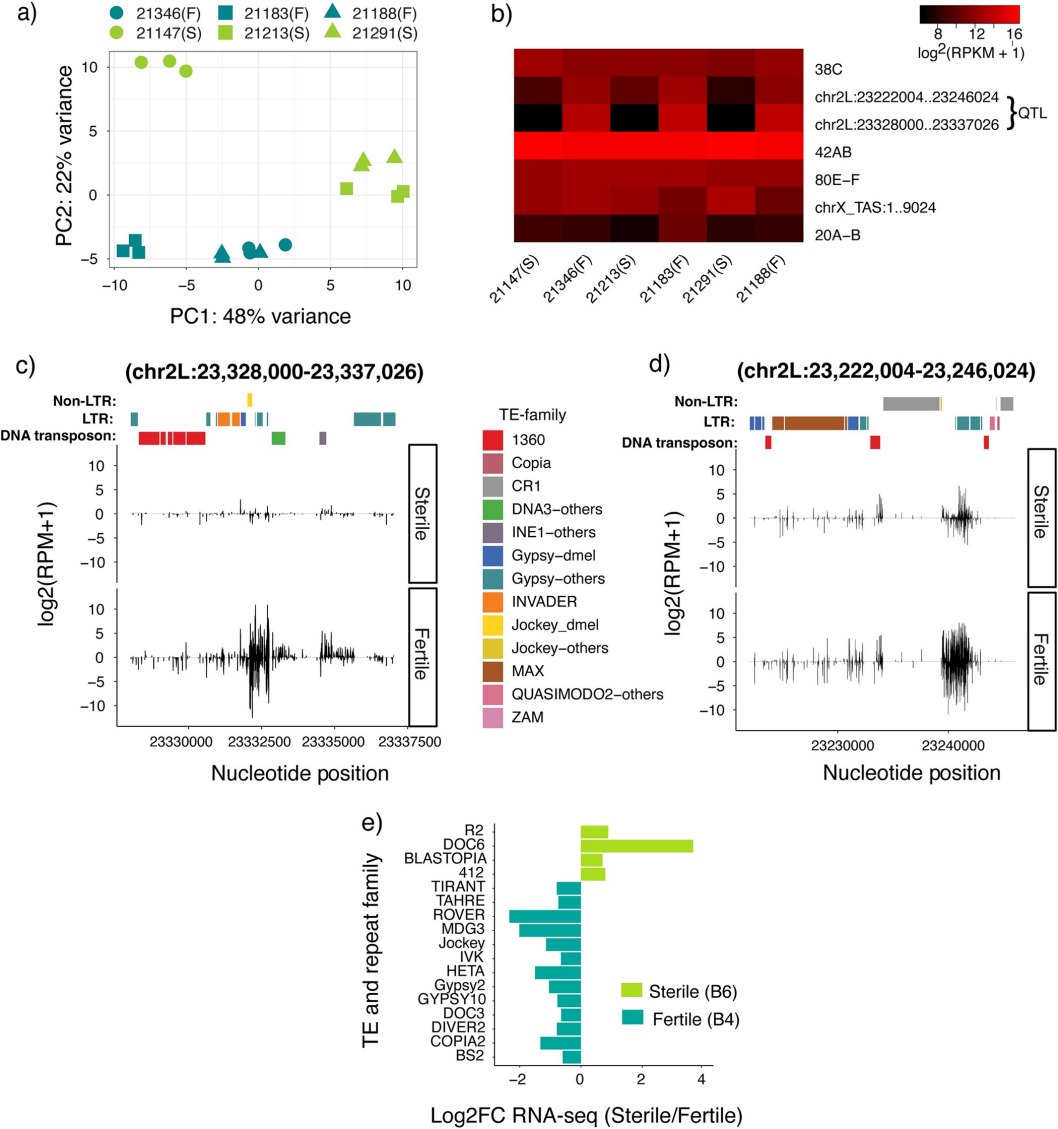
Dysgenic sterility is not related to differential activity of piRNA clusters or TE deregulation. **a**) PCA analysis for piRNA cluster expression data of sterile (S) and fertile (F) genotypes. Members of the same RIL pair are represented by the same shapes. **b)** Heat map showing the expression of seven major piRNA clusters [61] and the two differentially expressed QTL clusters in QTL-3d. RIL pairs are plotted adjacent to each other. **c and d)** Uniquely mapping piRNAs within two differentially active QTL-3d piRNA clusters are compared between sterile (21183) and fertile (21213) genotypes. Positive value indicates piRNAs mapped to the sense strand of the reference genome and negative value indicates those from the antisense strand. TE insertions in each cluster are presented according to family by different colors; TE-others indicate the insertion was most similar to a consensus TE from a sibling species of *D*. *melanogaster*. See **S2 and S3 Figs** for cluster expression in the remaining RIL pairs. For **b, c and d,** piRNA cluster expression levels are estimated by log2 scale transformed of reads per million mapped reads [log2(RPM+1)]. **e)** Genome-wide differences in TE family expression between sterile and fertile genotypes (fold change = 1.5, base mean > = 100, adjusted *p-*value < = 0.05), based on alignment to consensus sequences. The data used to plot panel **a** is provided in **S15 Table,** for panel **b** in **S8 Table**, for panels **c** and **d** in **S16 and S9 Tables**, and for panel **e** in **S10 Table**).

To look for differences in resident TE regulation, we performed small RNA-seq on the same ovarian samples from RIL females (mothers) that we used for total RNA-seq, and quantified the expression of piRNAs from clusters throughout the genome. A PCA of piRNA cluster expression reveals that sterile and fertile genotypes differ in the ovarian expression of some piRNA clusters, and are resolved by the second principal component, accounting for 22% variation in expression (**Fig 3A and S15 Table**). In particular, we discovered two small pericentromeric piRNA clusters located within QTL-3d that were active in fertile genotype ovaries but largely quiescent in sterile genotype ovaries (**Figs 3B, 3C, 3D,** **S2, and** **S3** **and S16 Table).** However, the major piRNA clusters—including *42AB*—do not differ in ovarian expression in between sterile and fertile alleles, suggesting that the proposed reduction in heterochromatin formation in sterile genotypes does not majorly reduce piRNA transcription (**Fig 3B and S8 Table**). Furthermore, the differentially active clusters in QTL-3d seem unlikely to regulate transpositionally active resident TE families, since they are largely composed of TE fragments that are relatively divergent from the consensus (65 to 95% sequence similarity; **Figs 3C, 3D,** **S2, and** **S3** **and S9 Table**), or are most similar to a consensus TE from other (non-*melanogaster*) *Drosophila* species. Transpositionally active TEs are generally highly similar to the consensus sequence [56], and piRNA silencing is disrupted by mismatches between the piRNA and its target [57]. Nevertheless, we cannot rule out the possibility that sterile or fertile genotypes could harbor TE insertions in this locus that are not represented in the *dm6* reference genome.

To directly address if fertile and sterile genotypes differ in resident TE regulation, we compared their genome-wide resident TE expression in our RNA-seq data. None of the TE families represented in the QTL-3d piRNA clusters were upregulated in sterile genotypes (**Fig 3E and S10 Table**). Furthermore, while some TE families are differentially expressed, there is no systematic increase in TE activity in the sterile genotypes. Rather, more TE families are upregulated in the ovaries of fertile genotypes (13 TEs) when compared to sterile (4 TEs) genotypes. Upregulation of certain TEs may be consistent with more late-stage egg-chambers in fertile genotype ovaries, since many retrotransposons accumulate transcripts in the oocyte over the course of oogenesis [58–60]. Therefore, despite the conspicuous position of QTL-3d surrounding piRNA producing-regions, as well as evidence for differential chromatin regulation that could impact piRNA biogenesis (**Fig 3B and 3E**), we find no evidence that fertility in dysgenic crosses is determined by resident TE silencing.

### Sterile alleles increase *P-*element mRNA expression and transposase mRNA splicing

Increased dysgenic sterility associated with sterile alleles could also reflect increased *P-*element transposition, resulting from increased *P-*element mRNA expression or splicing. In particular only transcripts in which the third intron (intervening sequence, IVS3) is spliced will produce P*-*transposase, and regulation of IVS3 splicing is a key determinant of *P*-element transposition [22]. We therefore examined the abundance of different *P-*element transcripts in the ovaries of F1 dysgenic offspring of sterile (B6) and fertile (B8) isogenic females. Dysgenic offspring in these experiments were reared at 22°C to avoid germline loss [20].

**Fig 4:**
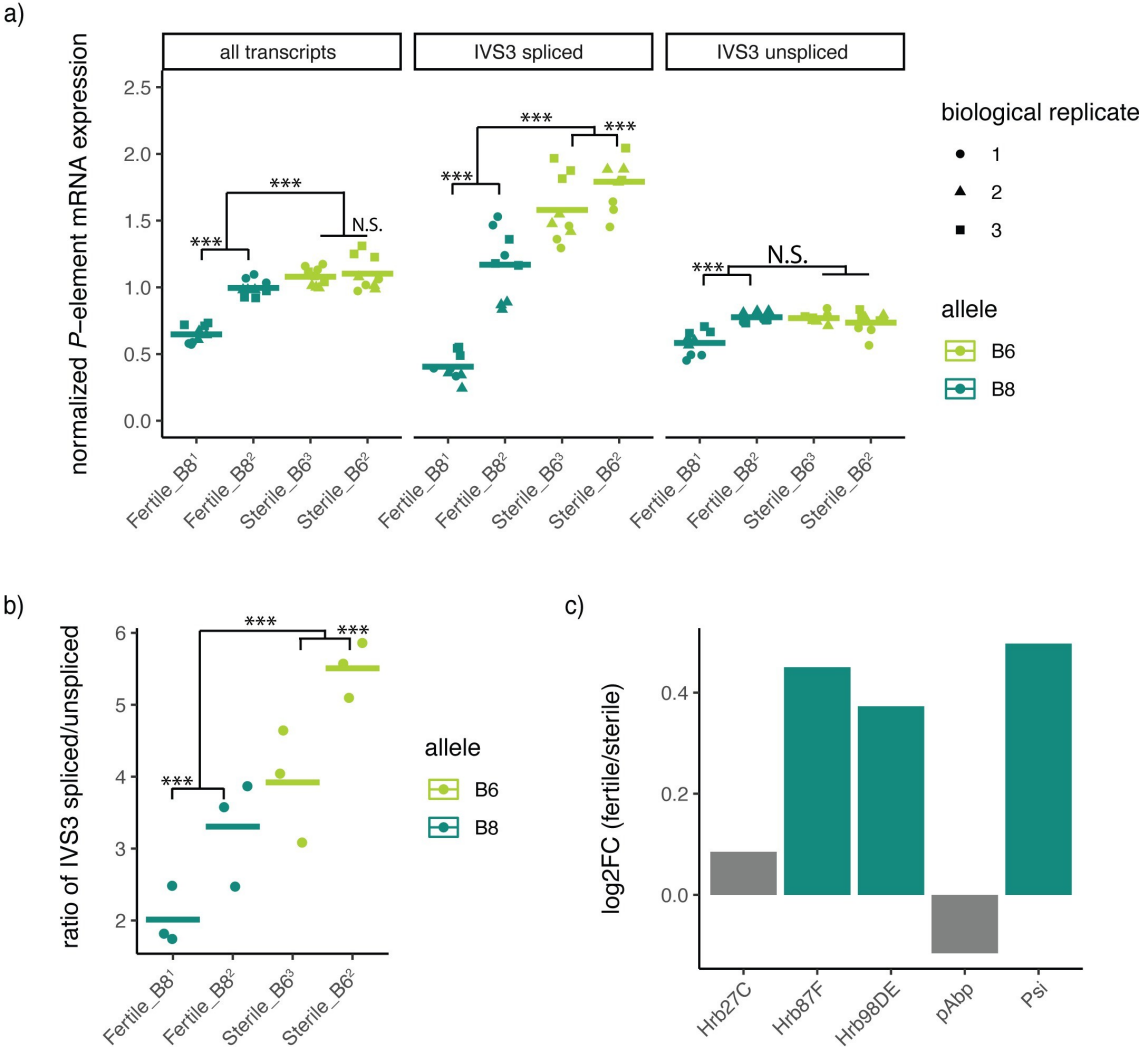
Decreased expression of P-transposase in fertile genotypes. A) Differential expression of *P*-element transcripts between fertile and sterile genotypes. Three separate qPCRs were performed, which detect all transcript isoforms, as well as IVS3 spliced and unspliced isoforms. qPCRs are normalized to rpl32. B) Ratios of IVS3 spliced to unspliced isoforms. C) Differential expression of splicing factors between sterile and fertile genotypes based on RNA-seq data. Dark green bars indicate factors that are significantly upregulated in fertile genetic backgrounds. Significant differences in qPCR data are based on linear models to detect differences between sterile and fertile genotypes, or Tukey-HSD comparisons to detect differences between genotypes containing the same allele. *** denotes P < 0.001.

Consistent with differential production of *P-*transposase, we observed differences in overall abundance of *P*-element transcripts between the F1 dysgenic offspring of sterile and fertile females (**Fig 4A,** *t10 =* 13.09, *p* = 2.31x10-15). On average, sterile females showed a 34% increase in *P-*element transcripts (95% CI 27–42%). Transposase-encoding (IVS3 spliced) transcripts show an even more pronounced 59% increase in expression in sterile genotypes (**Fig 4A,** *t10 =* 10.27, *p* = 2.91x10-10, 95% CI: 42–81%). By contrast, unspliced (IVS3 retaining) transcripts were not significantly differentially expressed between ovaries of F1 dysgenic offspring of sterile (B6) and fertile (B8) isogenic females (**Fig 4A,** *t10 =* 1.68, *p* = 0.11), although certain individual comparisons between strains were significant. To directly address whether splicing is more efficient in the ovaries of sterile dysgenic offspring, we compared the ratio of spliced to unspliced *P-*element transcripts (**Fig 4B**). The ratio of spliced to unspliced transcripts differed significantly between the ovaries sterile and fertile dysgenic offspring (*t10 =* 7.45, *p* = 7.30x10-5), suggesting that splicing itself is more efficient in sterile genotypes.

We also observed differences in splicing and expression between isogenic stocks carrying the same allele. In particular, the Fertile_B81 and Fertile_B82 differed in both spliced and unspliced transcripts, with a particularly pronounced 2.89-fold increase in spliced transcript expression in Fertile_B82 as compared with Fertile_B81 (**Fig 4A,** *t10 =* 12.14, *p* = 9.78x10-12, 95% CI: 2.61–3.17 fold). While the sample size is too small to draw any conclusions, it is notable that Fertile_B82 does not exhibit the same degree of fertility rescue as Fertile_B81, further pointing to a connection between spliced transcript production and dysgenic sterility (**Fig 1G and 1H**).

In germline cells, the splicing of IVS3 is known to be repressed by piRNA mediated transcriptional silencing, which is initiated by maternally transmitted piRNAs or through the production of *de novo* piRNAs in aged females [16,21]. In addition to the absence of maternally transmitted piRNAs, the splicing differences we observe here are in young (3–4 day old) dysgenic females, as opposed to aged dysgenic females, suggesting they are likely independent of the piRNA pathway. However, the splicing of IVS3 is also repressed by several host splicing factors in somatic cells, and it is proposed that some of these factors may also partially repress splicing in germline cells [62–64]. Consistent with piRNA-independent differences in splicing, we discovered that three splicing factors known to promote IVS3 retention in somatic cells, hrp36, hrp38 and *P*-element somatic inhibitor (Psi) show increased expression in fertile genotypes in our ovarian RNA seq data (**Fig 4C**). This suggests that decreased splicing in the dysgenic offspring of fertile isogenic lines may result from increased abundance of host splicing factors.

### Sterile alleles increase radiation sensitivity and accumulated mutations

Our gene expression data suggest that sterile and fertile alleles may differ in their capacity to repair germline DSBs in young (3 day) dysgenic females. Fertile alleles exhibit upregulation of the TIP60 complex (which is involved in DSB repair [34]), while sterile alleles exhibit upregulation of replication dependent histones (which may complete with DNA repair machinery [38]). Mutations in DSB repair genes are widely known to cause radiation sensitivity, which is easily quantified by measuring lethality following larval radiation exposure [65–69]. While this assay occurs in whole larvae as compared to female germlines, larvae are composed of numerous classes of mitotically dividing cells, similar to the primordial and premeiotic stages of gamete production in which *P-*element transposition occurs [16,21]. Furthermore, numerous key factors for germline DNA damage response, as well as germline *P-*element excision repair, exhibit larval radiation sensitivity phenotypes [70–75].

**Fig 5:**
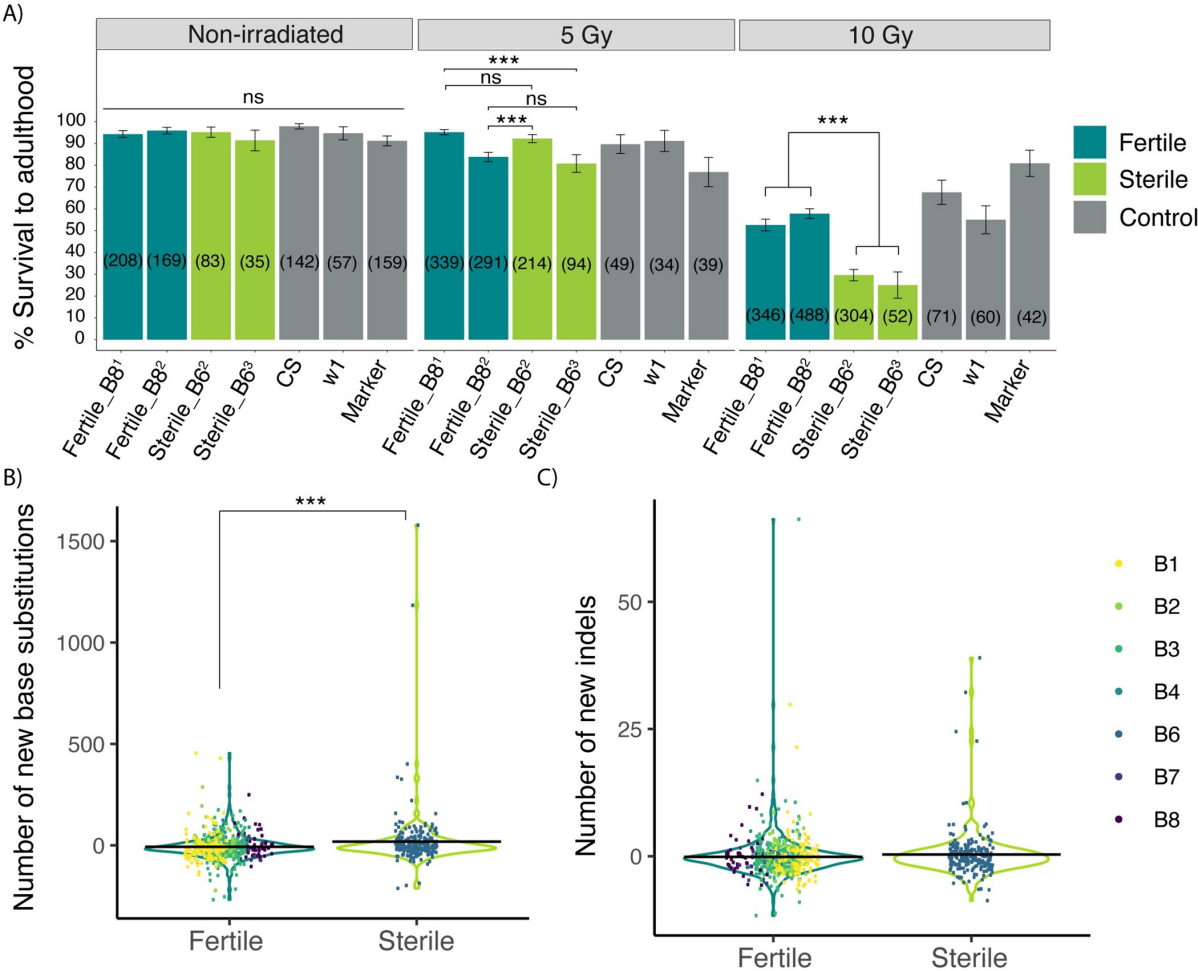
Sterile alleles exhibit reduced DNA repair. A) The percentage of mock treated and irradiated (5 Gy and 10 Gy) larvae that survived to adulthood for the fertile (B8), sterile (B6) and the control genotypes. CS refers to Canton-S and marker refers to the multiply marked stock *b cn* (#44229), which was used to generate isogenic lines. The numbers in the brackets refer to the sample size. The significance of comparisons between genotypes was determined by the χ2 test-of-independence. B and C) New mutations that accumulate in RIL genomes as detected by MSG. B) New SNPs and C) new indels. An excess of new mutations was detected by a t-test comparing Sterile B6 RILs to all others. The data represented in the Fig is provided in **S19 and S20 Tables)** ** denotes P< 0.01, *** denotes P < 0.001.

We therefore compared the X-ray radiation sensitivity of larvae from isogenic lines containing sterile (B6) and fertile (B8) alleles. Note these lines are the same as those in **Fig 1F–1H)**. After exploring a range of radiation doses, we found that doses above 10 Gy showed high lethality, making it difficult to detect differences in radiation sensitivity between the genotypes **(S19 Table)**. Therefore, we compared the response of sterile and fertile larvae to radiation doses of 0 Gy, 5 Gy and 10 Gy. We observed that fertile genotypes had significantly higher survival (53–58%) than the sterile genotypes (25–30%) at 10 Gy (**Fig 5A**). Given that X-ray radiation produces predominantly DSBs, these results are consistent with differences between fertile and sterile alleles in DSB repair.

We further looked specifically for differences in germline DSB repair by examining whether RILs carrying B6 alleles at the QTL-3d peak have accumulated more *de novo* base substitutions and small insertions or deletions. The DSPR RILs underwent 50 generations of inbreeding, and have since been maintained as isogenic lab stocks for ~175 generations, allowing ample time for new mutations to accrue as a consequence of deficient repair. To detect these new mutations, we generated multiplexed shotgun genotyping (MSG) libraries for 792 population B RILs [76]. This low coverage method (mean 2.9x) will uncover only a random subset of new mutations in each RIL, thereby underestimating the true amount of mutation accumulation. Nevertheless, we were able to detect 102,476 novel base substitutions and 5,026 novel insertions or deletions among the RIL MSG libraries.

After accounting for differences in sequencing depth and plate effects, the founder allele at QTL-3d was associated with differences in the number of new base substitutions (-2**Δ**lnL = 15.62, df = 6, p = 0.016). Furthermore, RILs carrying B6 alleles at the QTL-3d peak exhibit an average increase of 14.58 new base substitutions (95% CI 5.23–24.12), when compared to those carrying another founder allele (*t782* = 3.043, *P* = 0.0024, **Fig 5B**). In contrast, there was no significant association between founder allele at QTL-3d and new indels (-2**Δ**lnL = 1.37, df = 6, p = 0.97). Given the low coverage data as well the limited potential of short-read sequencing data to identify larger structural variation [77], we cannot be conclusive about a relationship between QTL allele and indel accumulation rate. Nevertheless, the increase in base substitution supports a deficiency in germline DNA repair in association with B6 alleles for QTL-3d.

### Identifying candidate genes

**Fig 6:**
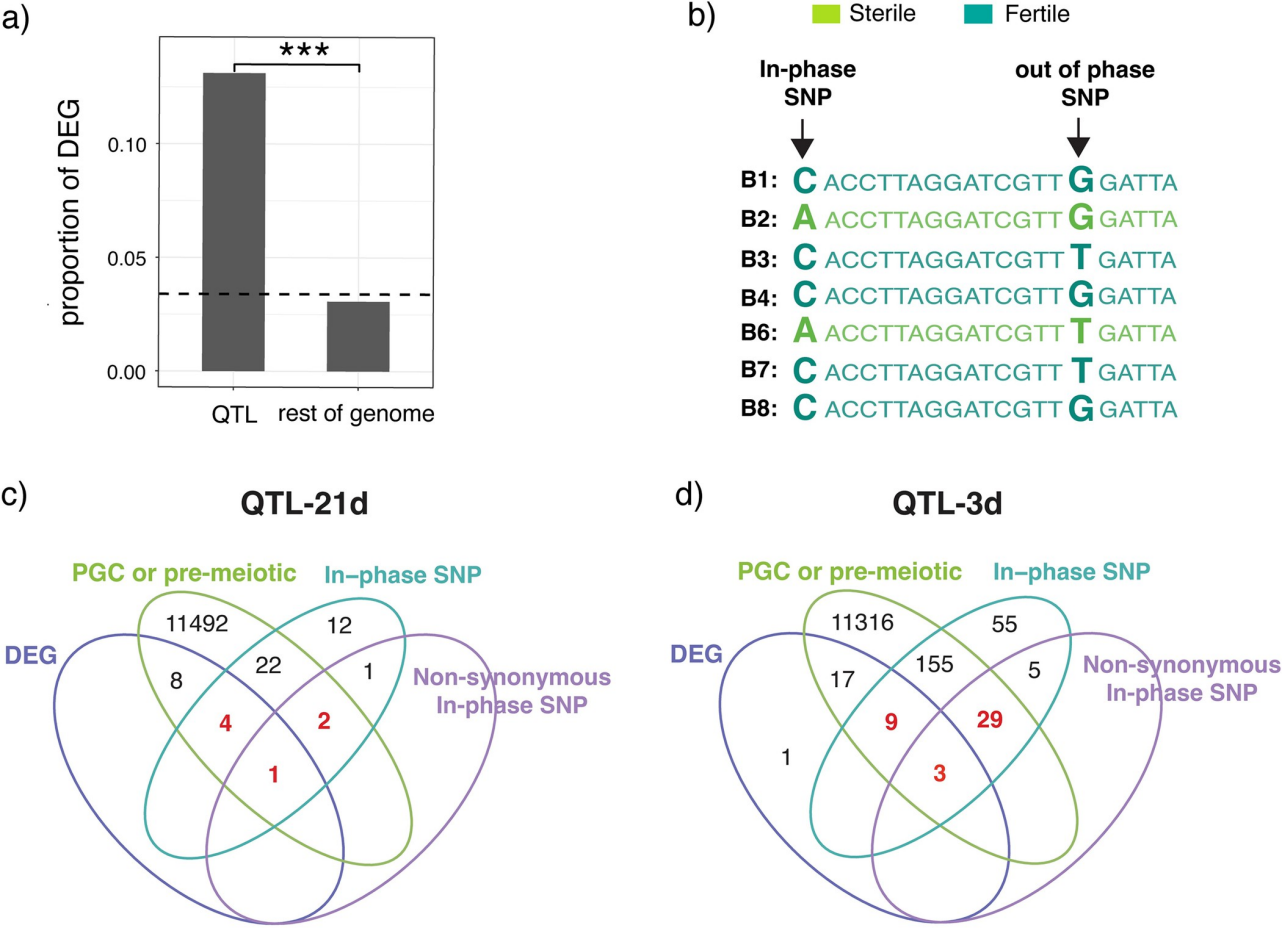
Differential expression and in-phase SNPs identify candidate genes. **a)** The proportion of genes differentially expressed (DEG) is compared inside and outside the QTL. The dotted line is the genome wide average. **b)** Hypothetical in-phase and out of phase SNPs are shown. Sequences of each of the B founder strains are colored based on their phenotypic classification, either fertile or sterile (**Fig 1E**). Bold letters indicate SNPs. **c and d)** Venn diagrams showing the overlap of differentially expressed genes (DEG), genes carrying in-phase synonymous and non-synonymous SNPs, and genes expressed in primordial or pre-meiotic germ cells for QTL-21d (c) and QTL-3d (d).The data for differential expression of genes for fertile and sterile genotypes is provided in **S5 Table**. The data on in-phase polymorphisms for each QTL peak are provided in **S11 Table**. List of candidate genes that have both in-phase polymorphisms and are differentially expressed, and those having non-synonymous in-phase polymorphisms are provided in **S12** and **S13 Tables**, respectively.

The QTL we map here are quite large and contain numerous candidate genes whose differential function could influence dysgenic sterility. Nevertheless, we next sought to identify candidate genes that influence dysgenic sterility for future study. We combined our own expression and mapping data with previously published polymorphism and single cell expression data to narrow candidates based on four criteria: 1) location within a QTL, 2) expression in primordial germ cells or early, pre-meiotic cysts [78,79], 3) differential expression between sterile and fertile adult ovaries, and 4) the presence of “in-phase” single nucleotide polymorphisms (SNPs) (**S11, S12 and S13 Tables**). In-phase SNPs are those where the genotypic differences between the founder alleles are consistent with their phenotype class (**Figs 1E and 6A** [80]). Of 530 differentially expressed genes, 43 are within the QTL region, representing an approximately five-fold enrichment in the QTL regions compared to the rest of the genome (χ2 = 255.54, *df* = 1, *p*-value < 2.2e-16, **Fig 6B**). Ultimately, we identified 12 and 5 differentially expressed genes and early germ cell expressed genes that also carry in-phase SNPs within the QTL-3d and 21d, respectively (**Fig 6C and 6D and S12 Table**). Furthermore, we identified 32 genes in QTL-3d and 3 genes in QTL-21d that exhibit early germ cell expression and also contain in-phase non-synonymous SNPs, which may affect the function of the encoded protein (**S13** Table). Collectively these genes represent the strongest candidates to contain causative variants.

We next scoured our list of candidate genes for those with known functions in chromatin regulation, DSB repair, or alternative splicing, whose differential function or regulation are plausibly related to the phenotypic differences associated with sterile and fertile alleles. None of the three splicing factors we discovered are differentially expressed reside within the QTL (**Fig 4C**), suggesting their expression differences arise as a consequence of regulatory differences in *trans*. While we did not discover any transcription factors located in the QTL that are differentially expressed in fertile and sterile ovaries, we did discover three C2H2 zinc finger transcription factors, *tio* and *CG30431* (QTL-3d) and *CG17568* (QTL-21d), that are located within QTL and contain in-phase non-synonymous SNPs. Unfortunately, the genomic binding sites of these transcription factors are undetermined, so it remains unknown if they are regulators of *hrp36*, *hrp38*, or *psi* transcription.

With respect to differences in chromatin state and/or DNA repair, two genes within QTL-3d, stand out as particularly attractive candidates; *Nipped-A* and *jing*. *Nipped-A* contains a non-synonymous in-phase SNP and is expressed in both PGCs and in germline cells throughout the earliest stages of oogenesis (**S13 Table**). *Nipped-A* is a member of the TIP60 complex, which has functions in DSB repair, chromatin modification and chromatin remodeling. Additionally, we identified multiple TIP60 components upregulated in fertile ovaries (**Fig 2C**). The non-synonymous SNP that separates sterile and fertile alleles of this gene are located in the HEAT2 domain, which is predicted to be essential for protein-protein interaction, and could have important implications for the function of this multi-protein complex [81–83]. *Jing* contains in-phase synonymous and non-synonymous SNPs, is upregulated in fertile ovaries, and exhibits a similar expression pattern in germline cells to that of *Nipped-A* (**Fig 2**E and **S12 and S13 Tables**). Based on a yeast-two hybrid screen Jing physically interacts with inverted repeat binding protein 18 (IRBP18): a DNA binding protein that comprises part of a heterodimer that binds directly to *P*-element’s transcribed inverted repeats, and facilitates repair of donor DNA after excision [84,85]. Furthermore, *irbp18* mutants exhibit larval radiation sensitivity, similar to our sterile genotypes (**Fig 5A**). If Jing determines differential activity of IRBP18 it could have a strong impact on dysgenic sterility. Beyond this function, Jing acts as an important cofactor of polycomb repressor complex 2, many of which showed increased expression in fertile ovaries Fig 2C, [86,87].

## Discussion

**Fig 7:**
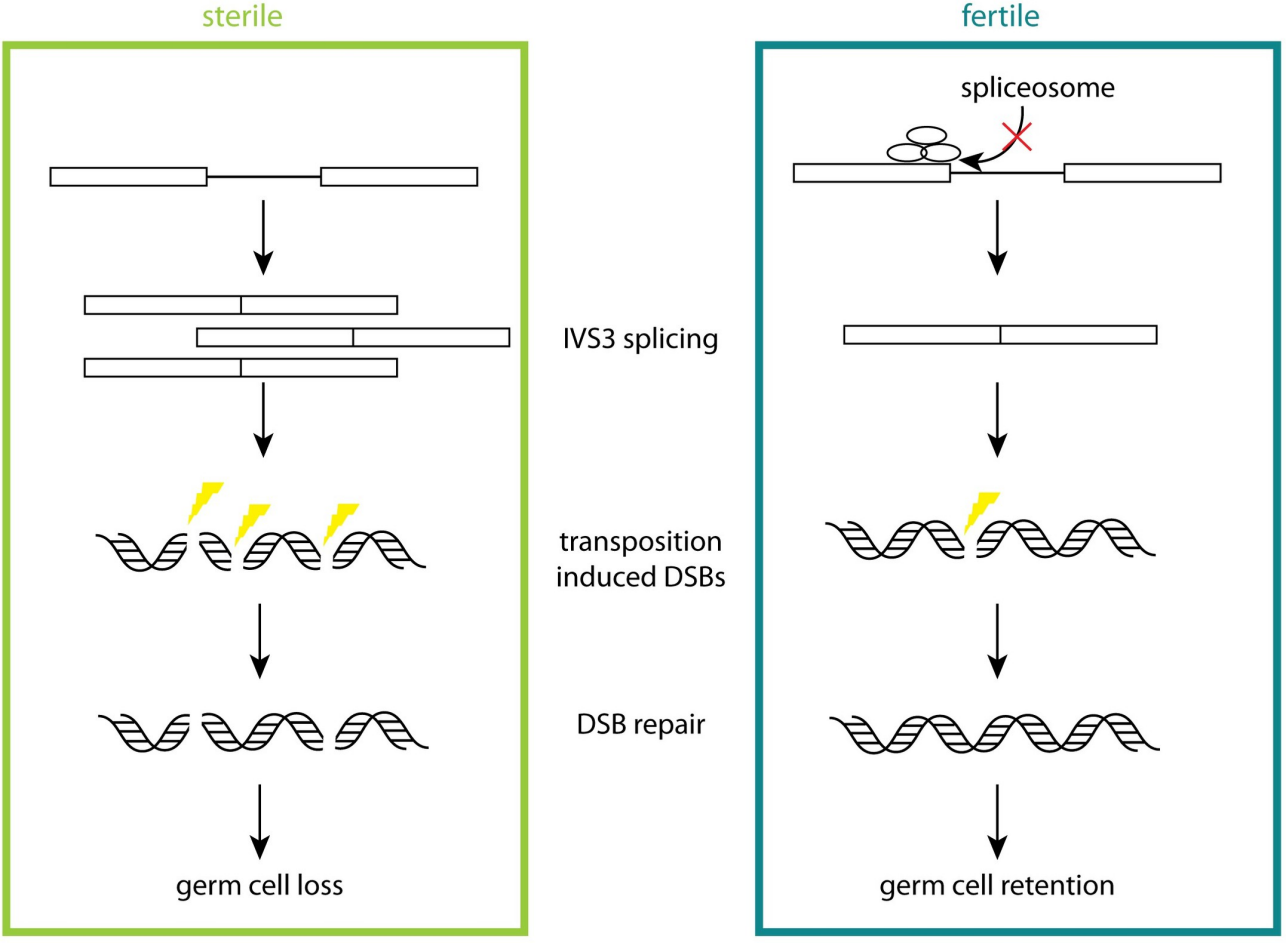
Schematic of phenotypic differences between sterile and fertile alleles. Differences in sterile and fertile alleles between IVS3 splicing and DNA repair are represented. In fertile alleles, host splicing suppressor expression is increased, leading to reduced production of spliced P-transposase encoding transcripts. As a consequence of reduced transposase production, it is predicted that fewer DSBs are produced in dysgenic females. However, it is also predicted that those breaks that are produced are repaired more efficiently.

Although small RNA mediated TE regulation is widely studied, little is known about genetic variation in host factors that modulate the germline transposition of invading TEs and their associated fertility effects. Here we uncovered natural variation in dysgenic sterility imposed by *P-*element DNA transposons. Our work points to two major differences between sterile and fertile genotypes, which likely explains the differential occurrence of dysgenic sterility between them (Fig 7). First, fertile alleles suppress the splicing of transposase-encoding mRNA, which likely reduces the occurrence of germline DSBs that drive germ cell loss. Second, fertile alleles are more tolerant of DSBs, perhaps due to enhanced repair, which may allow them to retain germ cells despite the genotoxic effects of transposition. We propose that these differences highlight two axes of host-TE interaction: permissivity and tolerance.

### Host splicing factors determine differences in permissivity

As intracellular parasites, TEs rely on host machinery for transcription, translation and replication. Variation in host co-factors that modulate these processes could drive differences in permissivity to TE proliferation. The concept of permissivity is prevalent in virology, and refers to the degree to which an individual cell type allows a virus to replicate [88–90]. With respect to TEs, permissivity is distinguishable from repression in that host-cofactors modulating permissivity precede the introduction of a TE into the genome, and the primary function of permissivity factors is not in TE regulation. For example, it has long been known that *P-*elements do not transpose in somatic cells due to the presence of host splicing factors that prevent the correct splicing of *P-*transposase encoding mRNA [22,63,91]. While the primary function of these splicing factors is not to regulate *P-*elements, their expression renders somatic cells non-permissive.

In germline cells, maternally deposited piRNAs also regulate *P-*element transposition by promoting IVS3 retention [16]. However, even in the absence of maternally deposited piRNAs, IVS3-retaining transcripts are common, suggesting other host factors may also modulate *P-*element splicing in the germline [16,64]. Our work here reveals that there is host genetic variation in IVS3 splicing that is independent of maternally deposited piRNAs, which has potentially dramatic impacts on host fitness in dysgenic crosses (**Figs 1B, 1G, 1H and 4A**). While we cannot completely rule out a potential role for *de novo* piRNA production in driving these splicing differences, their occurrence in young dysgenic females and the absence of strong age effects on dysgenic sterility in our experiment points away from this explanation. Rather, the upregulation of multiple host splicing regulators in fertile genotypes suggest that the same factors that regulate IVS3 splicing in somatic cells may also modulate splicing in germline cells (**Fig 4C**). While we did not directly address whether these differences lead to differential transposition of *P-*transposons, these results suggest that fertile genotypes, like somatic cells, reduce permissivity through splicing regulation.

#### Tolerance and DSB repair

In our previous work on natural variation in dysgenic sterility, we proposed that host genotypes may differ in tolerance: the ability of germline cells to persist and divide despite the damaging effects of transposition [14]. Because hybrid dysgenesis occurs through the loss of larval primordial germ cells and adult germline stem cells [15,16], the variable expression of factors that determine stem cells maintenance and differentiation could be an important source of tolerance variation. In particular, we found that the function of *bruno*, a differentiation factor in early pre-meiotic cysts, increases dysgenic sterility [14]. Conversely, the overexpression of the stem cell factor *myc* in dysgenic larval PGCs suppresses their loss, and by association, decreases dysgenic sterility [17].

Our work here suggests that genetic variation in DNA damage response or repair provides another potential mechanism of tolerance. Since transposition results in DSBs at sites of insertion and excision, enhanced ability to detect and repair these breaks would help reduce dysgenic germ cell loss. We observed that fertile genotypes are significantly more resilient to X-ray radiation (**Fig 5A**), a phenotype that is widely associated with increased activity of DNA repair genes [65–69]. Indeed, the magnitude of the differences in radiation sensitivity is large, and mirrors previous comparisons between wild-type and DNA damage response mutants such as *p53* or *checkpoint kinase 1* [74,75]. We further observed that fertile genotypes exhibit fewer accumulated base substitutions (**Fig 5B**), suggesting heritable differences in DNA repair. While DNA damage signaling is a clear determinant of dysgenic germ cell loss [15,18,19,21], to our knowledge this is the first evidence that natural variation in DNA repair may modulate the sterility effects of transposition. However, we cannot rule out the possibility reduced DSB repair also increases permissivity, by prolonging S-phase thereby allowing more time for *P-*elements to transpose.

Something that remains puzzling about our observations regarding DNA damage and repair is that it is not intuitively obvious how deficiencies in DSB repair would lead to an accumulation of base substitutions in RILs carrying sterile alleles. However, DNA repair pathways are interdependent, with many components impacting multiple repair processes. For example, *mei-9* is required for both nucleotide excision repair and meiotic recombination [92], and mutant alleles are known to enhance germline loss in *P*-element dysgenic males [18,93]. Beyond this, homologous repair of DSBs leads to an increased rate of base substitution, potentially due to the sensitivity of single stranded repair intermediates to other forms of DNA damage reviewed in [94]. Therefore, if the DSB repair deficiency associated with sterile alleles results in a larger number of DSBs repaired by homologous pathways, or a delay in those repairs, an increase in base substitution is predicted.

## Conclusion

The degree to which innate differences among hosts govern the propagation of an invading TE, as well as its fitness effects during invasion, is an understudied aspect of TE invasion biology. Here we have uncovered two different forms of host genetic variation in dysgenic sterility, which alter the permissivity of host cells to transposition, as well as their tolerance to transposition’s effects. These observations add complexity to our current understanding of how host genetic variation can modulate the fitness effects of an invading TE. The precise pathways and genetic factors whose differential function underlie these tolerance and permissivity phenotypes remain to be resolved. Similarly, the degree to which these processes reflect the pleiotropic effects of a single gene, or the combined action of multiple factors remains an important question to be addressed by future work.

## Methods

### *Drosophila* strains and husbandry

The recombinant inbred lines are described in King *et al*. [24]. Harwich (#4264) and *b cn* (#44229), were obtained from the Bloomington *Drosophila* stock center. Canton-S was obtained from Brigitte Dauwalder. All flies were maintained on standard cornmeal media.

Alleles of the second chromosome centromeric region, containing both QTL, were extracted from three recombinant inbred lines carrying B6 QTL allele (#21076, #21218, #21156) and two RILs carrying B8 QTL allele (#21077, #21154) into a common background by crossing them to multiply marked stocks *b cn* (#44229). After 7 rounds of backcrossing followed by inbreeding, the final isogenic lines (Sterile_B61, Sterile_B62, Sterile_B63 and Fertile_B81, Fertile_B82) were generated. The lines were made homozygous for the 2nd chromosome by inbreeding and selecting for wild type phenotype. The genotype of the isogenic lines were verified through PCR using five different primers within the two QTL. chr2L:19383155–19383970: AACCCTTTTTCGCTGACAATAACA, ATTATCAGCAGGAGCCGGAAACTT; chr2L:21333500–21334300: AAGTGAAGCTAACAACGTGACAAC, CGTTTGACCATCGCTTACAACTAA; chr2R:2392800–2393600: AACAGGAGGTCGAAAGCCAAATA, ATGCAGAGTCATATTCTGGGTTGG; chr2R:6203290–6204284: AATGGAGACCGTTGATTTTGGTAA, CTTTTCTGCGGCATCAGGTG; chr2R:6058000–6059000: TGGCAATTGCAATCCTTTTGGTAT, ATAACACGAACTACGACCTTTCCA.

#### Phenotyping

Phenotyping of ovarian atrophy was performed as described previously in Kelleher *et al*. [14]. Briefly, crosses between virgin RIL females and Harwich males were transferred to fresh food every 3–5 days. Since crosses reared at a restrictive temperature (29oC) result in complete gonadal atrophy in F1 offspring, we reared our crosses at a lower permissive temperature (25oC), which produces an intermediate phenotype that better reveals the variation in severity of dysgenesis [12,14,15,95]. F1 offspring were maintained for 3 days or 21 days, at which point their ovaries were examined using a squash prep [95]. 21 day- old females were transferred onto new food every 5 days as they aged to avoid bacterial growth. Females who produced 1 or more chorionated egg chambers were scored as having non-atrophied ovaries, and females producing 0 egg chambers were scored as having atrophied ovaries.

Crosses and phenotyping were performed for 673 RILs across 22 experimental blocks for 3 day-old F1 females, and 552 RILs across 18 experimental blocks for 21 day-old F1 females. If fewer than 21 F1 offspring were phenotyped for the same cross, it was discarded and repeated if possible. In total, we phenotyped >20 3-day old and 21 day-old F1 female offspring for 595 RILs and 456 RILs, respectively.

#### QTL mapping

QTL mapping was performed as described in Kelleher *et al*. [14]. Briefly, for each developmental time point, we modeled the arcsine transformed proportion of F1 ovarian atrophy as a function of two random effects: experimental block and undergraduate experimenter. Regression models were fit using the lmer function from the lme4 package [96]. We then used the residuals as a response for QTL mapping with the DSPRqtl package [24] in R 3.02 [97]. The LOD significance threshold was determined from 1,000 permutations of the observed data, and the confidence interval around each LOD peak was identified by a difference of -2 from the LOD peak position (**Δ**2-LOD) [26], or from the Bayes Confidence Interval [98]. For **Δ**2-LOD intervals, we took the conservative approach of determining the longest contiguous interval where the LOD score was within 2 of the peak value. We further calculated the broad sense heritability of ovarian atrophy as in Kelleher *et al*. [14].

#### Estimation of founder phenotypes and QTL phasing

To estimate the phenotypic effect associated with each founder allele at the QTL peak, we considered the distribution of phenotypes from all RILs carrying the founder haplotype at the LOD peak position (genotype probability >0.95%) [24]. QTL were then phased into allelic classes by identifying the minimal number of partitions of founder haplotypes that describe phenotypic variation associated with the QTL peak, as described previously [14,24].

#### Fertility assays

Virgin female offspring from dysgenic crosses between isogenic lines carrying fertile_B81/B82 (21077, 21154) and sterile_B62/B63 (21218, 21156) alleles and Harwich males were collected daily and individually placed in a vial containing two Canton-S males. Females were allowed to mate for 5 days and were transferred to a new vial for another 5 days after which the parents were discarded. The presence and total number of F2 individuals were counted from the two vials.

#### Selection of paired RILs with alternate QTL alleles

We identified background matched RILs containing either the B6 (sterile) or B4 (fertile) haplotypes from the start position of the QTL-21d confidence interval (2L: 19,010,000) to the end position of QTL-3d confidence interval (2R: 6,942,495) (*P* > 0.9), based on their published HMM genotypes [24]. For all possible RIL pairs (B6 and B4), we then calculated the number of 10 Kb genomic windows in which they carried the same RIL haplotype (*P* > 0.9). We selected three pairs of RILs, which carry the same founder genotype for 47% (21213 & 21183), 46% (21147 & 21346) and 44% (21291 & 21188) of genomic windows outside of the QTL.

#### Small RNA-seq and total RNA-seq

RILs were maintained at 25°C, and three biological replicates of 20 ovaries were dissected from 3–5 day-old females. Ovaries were homogenized in TRIzol (invitrogen) and stored at -80°C until RNA extraction. 50 μg of total RNA from each of 18 biological samples (3 biological replicates x 3 pairs) was size fractionated in a 15% denaturing polyacrylamide gel and the 18–30 nt band was excised. 2S-depleted small RNA libraries for Illumina sequencing were then constructed according to the method of Wickersheim and Blumenstiel [99]. Ovarian small RNA libraries were published previously SRP160954, [100]. Ribodepleted and stranded total RNA libraries were generated from the same ovarian samples using NuGen total RNA kit (TECAN). All 18 small RNA and total RNA libraries were sequenced on an Illumina Nextseq 500 at the University of Houston Seq-N-Edit Core.

#### Small-RNA analysis

Sequenced small RNAs were separated based on size into miRNAs/siRNAs (18-22nt) and piRNAs (23-30nt) [11]. Reads corresponding to contaminating rRNAs, including 2S-rRNA, were removed from each library by aligning to annotated transcripts from flybase [101]. To determine the piRNA cluster activity we first uniquely aligned the piRNAs to reference genome (dm6 [29]) using Bowtie1 (-v 1 -m 1) [102]. We then used a customized perl script to count reads that mapped to a set of previously annotated piRNA clusters from the same genotypes (497 piRNA clusters, [103]). Read counts normalized to total mapped microRNAs for each library were used to infer differential expression using DESeq2 [104]. Sliding window estimates of piRNA abundance (**Fig 2C and 2D)** were calculated using bedtools genomecov [105], normalizing the read counts to total mapped miRNA reads.

#### Total RNA analysis

Residual ribosomal RNAs (rRNAs) were identified in ribo depleted libraries based on alignment to annotated rRNAs from flybase [101], and excluded from further analysis. Retained reads aligned to the library of consensus satellite and TE sequences from repbase [106], plus additional satellite consensus sequences from Larracuente [107]. For TE expression, the total reads mapped to TE sequences were counted using unix commands (uniq -c). Remaining reads that failed to map were pseudoaligned to *D*. *melanogaster* transcriptome (dm6/BDGP6) using Kallisto with default parameters [108]. Differentially expressed TEs and genes were identified from a combined analysis in DESeq2 [104]. Genes and TEs with base mean > = 100, Adjusted *P*-value < = 0.05 and whose expression pattern differed (fold change > = 1.5) were considered differentially expressed between the B6 and B4 QTL haplotype.

#### Radiation sensitivity

Third instar larvae were either mock treated or irradiated in a Rad Source RS 1800 X-ray machine set at 12.5 mA and 160 kV. To obtain 3rd instar larvae, embryos were collected for 24 hr and aged for 5 days at 25°C. The food vials containing larvae were then X-ray irradiated at doses from 5–80 Gray after which an optimal dose that clearly depicts the phenotypic difference was selected. Survival to adulthood was determined by scoring the number of empty and full pupal cases at 10 days after radiation.

#### Identification of novel mutations in RIL genomes

10 females from each of 795 RILs were deposited into a well of a 96 well plate (Axygen, P-96-450R-C) on ice. DNA isolation was then executed in plates using the Gentra Puregene Cell Kit (Qiagen, 158788) using extensions of the manufacturer’s protocol. Subsequently, DNA was further purified (Qiagen, QIAquick 96 PCR Purification kit, 28183), quantified via a fluorometer (ThermoFisher, Qubit dsDNA HS kit, Q32854), and diluted to 2-ng/μl. Each RIL sample was then subjected to the MSG (Multiplexed Shotgun Genotyping) approach developed by Andolfatto *et al*. [76], which is a form of RADseq (restriction-site associated DNA sequencing, Baird *et al*. [109]). Starting with 10-ng DNA, samples were first digested using MseI (New England Biolabs, R0525L). This enzyme has the restriction site T/TAA, cuts frequently along the genome, and unlike traditional rare-cutter RADseq strategies, yields sequencing reads spread somewhat evenly along the genome. Next, plate well-specific barcoded adapters are independently ligated onto the cut ends of each DNA sample. Fragmented, barcoded samples from a given plate are then mixed, and the 96-plex pool precipitated, purified, and size-selected to 250-300-bp via a Pippin Prep (Sage Science). Each multiplexed sample is then PCR amplified, during which a DNA plate-specific Illumina-compatible index is incorporated, and then purified. Finally, each of the independently-indexed 96-plex pools are quantified, mixed at equal concentration, and sequenced over multiple lanes of an Illumina HiSeq 2500 on “high output” mode, yielding single-end 100-bp reads (KU Genome Sequencing Core). With this MSG approach, reads from each RIL are computationally distinguished by both an Illumina index sequence (which marks the plates), and an “in line barcode” (the first 6 Read1 bases, which marks samples on any given plate).

SNP and indel variants were identified from MSG short-read data following GATK best practices for sample groups [110–112]. Briefly, Cutadapt (version 3.5; [113]) was used to de-multiplex samples and trim adaptors, while alignment to the *D*. *melanogaster* reference genome (dm6 [29]) were performed using BWA (version 0.7.17-GCC-10.2.0;[114]). The resulting BAM files were sorted and indexed using Samtools (Li et al., 2009 [114]). Individual GVCF files were generated using HaplotypeCaller and then joint-genotyped using Genotype_GVCFs. Both SNPs and indels were extracted and filtered out following the GATK Best Practices hard filters using VariantFiltration (SNP: "QD < 2.0 || FS > 60.0 || MQ < 40.0 || MQRankSum < -12.5”; indel “"QD < 2.0 || FS > 200.0”, and converted to TSV in R for further analysis using the vcfR package [115].

To identify novel base substitutions that arose in RILs and not present in the founders, we filtered out all alleles with spanning deletions as well as annotated SNPs from the founder lines (https://wfitch.bio.uci.edu/~dspr/Data/index.html). Since founder SNPs were called in dm5, we converted their coordinates to dm6 using the NCBI Genome Remapping Service (https://www.ncbi.nlm.nih.gov/genome/tools/remap) before filtering. For indels, short indels were not called from the original founder sequence data. We therefore considered an indel to be novel if they were unique to a RIL and sequenced among at least 50 RILs.

Differences in the number of SNPs and indels rate was modeled using linear mixed model fitted with lmer function in the R package lme4 (Bates et al., 2013 [96]). Three models were compared, a null model (mutations ~ plate + depth), a founder allele model (mutations ~ plate + depth + founder allele), and a dysgenic sterility allele model (mutations ~ plate + depth + sterile/fertile). Founder or dysgenic sterility allele referred to the QTL-3d peak: (B1-B5,B6-B8) or sterile/fertile (B6/all other founders). Note that B5 alleles are not present among the RILs at the QTL-3d peak. The models were compared using a likelihood ratio test to determine whether founder allele or allelic class explained variation in the number of novel SNPs or indels between RILs. The effect of sterile alleles on SNP and indel number was evaluated by *t*-test.

### qRT-PCR of P-element transcripts

3 biological replicates including 20 pairs of 3–5 day F1 dysgenic ovaries from crosses between fertile (B8^1^, B8^2^) or sterile (B6^2^, B6^3^) females and Harwich males were dissected and homogenized in TRIzol. Crosses were maintained at 22°C. RNA was treated with DNA-free (ThermoFisher) and reverse transcribed using oligo-dT primers and superscript IV (ThermoFisher) according to manufacturer instructions. Three different primer sets were used to amplify the 3’ end of all *P*-element transcripts, IVS3 spliced transcripts and IVS3 unspliced transcripts, as well as rpl32. Transcripts were amplified and quantified in three technical replicates using power-SYBR green (ThermoFisher) and normalized to rpl32 for the same sample.

Primer sequences were as follows: rpl32-F: 5’-CCGCTTCAAGGGACAGTATC, rpl32-R: 5’-GACAATCTCCTTGCGCTTCT, P-element-all F: 5’-CACCGAAATGGATGAGTTGACG, P-element-all R: 5’-TAATAAGTCCGCCGTGAGACAC, P-element IVS3 spliced F: 5’-AATAGCCAGGAATACAGAAATG, P-element IVS3 spliced R: 5’-AACATTTCTGTATTCCTGGCTA, P-element IVS3-unspliced F: 5’-GACAAAACACAATAGACAGCACA, P-element IVS3-unspliced R: 5’-TGTGCTGTCTATTGTGTTTTGTC.

#### Identification of in-phase polymorphisms

The SNP data of B founders that used to infer in-phase SNPs is based on *dm3* [24]. To identify in-phase SNPs we looked for alternate SNP alleles that match the predicted phenotypic class for each of the QTL peaks. For QTL-21d we used the criteria: sterile class (B2, B6) and the fertile class (B1, B3, B4, B7, B8), whereas for QTL-3d: sterile class (B6) and the fertile class (B1, B2, B3, B4, B7, B8).

## Supporting information

S1 Fig Sterility is associated with increased expression of pericentromeric genes in the head. a) Mean expression of genes located in the pericentromere, euchromatin, telomere and the fourth chromosome from RILs carrying each of the eight B founder genotypes at the QTL-3d region. Error bars represent the standard deviation among mean expression levels of different genes. The sterile/B6 (light green) shows high pericentromeric gene expression compared to the fertile strains (dark green) (Anova; F6,494 = 7.775, P < 5.24e-08). The letters indicate significantly different expression levels based on Tukey-HSD comparisons between RILs with different founder alleles.
(PDF)

S2 Fig Expression profile of QTL piRNA clusters in sterile and fertile RIL pair 2. The piRNA expression between sterile and fertile genotypes from the 21188–21291 RIL pair along the two QTL piRNA clusters: 2L:23,328,000–23,337,026 and 2L:23,222,004–23,246,024, respectively. Only uniquely mapping piRNAs are considered. The TE families at the top of each panel are represented by different colors. TE-others represent the repeat families coming from sibling species of *D*. *melanogaster*. Positive value indicates piRNAs mapped to the sense strand of the reference genome and negative value indicates those from the antisense strand. The piRNA cluster expression levels are estimated by log2 scale transformed of reads per million mapped reads [log2(RPM+1)].
(PDF)

S3 Fig Expression profile of QTL piRNA clusters in sterile and fertile RIL pair 3. The piRNA expression between sterile and fertile genotypes from the 21346–21147 RIL pair along the two QTL piRNA clusters: 2L:23,328,000–23,337,026 and 2L:23,222,004–23,246,024, respectively. Only uniquely mapping piRNAs are considered. The TE families at the top of each Fig are represented by different colors. TE-others represent the repeat families coming from sibling species of *D*. *melanogaster*. Positive value indicates piRNAs mapped to the sense strand of the reference genome and negative value indicates those from the antisense strand. The piRNA cluster expression levels are estimated by log2 scale transformed of reads per million mapped reads [log2(RPM+1)].
(PDF)

S4 Fig Sterility among 3 and 21 day females based on QTL haplotype. Four haplotypes are compared, which comprise all possible combinations of sterility alleles at 2 QTL. The allele at the 3 day QTL is indicated first and is represented by the color of the violin plot (light green = sterile, dark green = fertile). The allele at the 21 day QTL is indicated second and represented by the color of the points on the scatter plot. Y-axis is residual variation in F1 atrophy after accounting for student experimenter and block. Among 3 day old females, haplotypes containing different alleles for the 3 day old QTL are significantly different from each other (Tukey HSD P = 0.016–0). However, haplotypes containing alternative QTL for the 21d only do not differ from each other (Tukey HSD P>0.74). This suggests phenotypic variation in 3 day old females is not influenced by their genotype at the 21 day QTL. In contrast, among 21 day old females tolerant alleles in both QTL loci are required to significantly decrease sterility below the sterile allele containing haplotypes (Tukey HSD P = 0.01–0).
(PDF)

S5 Fig Crossing scheme to generate sterile and fertile alleles. (PDF)

S6 Fig Increased expression of genes upregulated in late-stage egg chambers in fertile ovaries. Upregulation in stage 9–10 and stage 12–14 egg chambers is from Tootle *et al*. [30]. Genes are separated into eggshell components (top) and non-eggshell components (bottom). Dark green bars indicate genes significantly upregulated in fertile genotypes whereas light green indicates genes upregulated in sterile genotypes.
(PDF)

S1 Table Provided are the proportion of atrophy for 3-day old F1 females when recombinant inbred lines were crossed to Harwich males. (XLSX)

S2 Table Provided are the proportion of atrophy for 21-day old F1 females when recombinant inbred lines were crossed to Harwich males. (XLSX)

S3 Table Residuals from 3-day-old F1 females used for QTL mapping. (XLSX)

S4 Table Residuals from 21-day-old F1 females used for QTL mapping. (XLSX)

S5 Table Results of DESeq2 analysis of differential gene expression between sterile and fertile alleles. (XLS)

S6 Table List of differential expressed of Tip60 members and one of its interactors. (XLS)

S7 Table List of genes upregulated in sterile and fertile ovaries, as well as associated enriched GO terms. (XLS)

S8 Table Analysis of piRNA cluster expression and abundance of P and I element derived piRNAs in sterile and fertile ovaries. (XLS)

S9 Table TE composition of differentially active piRNA clusters in QTL-3d. (XLSX)

S10 Table Results of DESeq2 analysis of differential TE expression between sterile and fertile alleles. (XLS)

S11 Table In phase polymorphisms in QTL-3d and QTL-21d. (XLSX)

S12 Table List of candidate genes that are differentially expressed between sterile and fertile alleles and contain non-coding in-phase SNPs. (XLSX)

S13 Table List of candidate genes that are contain non-synonymous in-phase SNPs. (XLSX)

S14 Table PCA analysis of gene expression data of background-matched recombinant inbred lines. (XLS)

S15 Table PCA analysis of piRNA cluster expression data of background-matched recombinant inbred lines. (XLSX)

S16 Table Uniquely mapping read coverage of piRNAs from sterile and fertile ovaries for both plus and minus strand of differentially active piRNA clusters in QTL-3d. (XLSX)

S17 Table Incidence of ovarian atrophy among in F1 females from crosses between isogenic Fertile/Sterile lines and Harwich males. (XLSX)

S18 Table Fertility of F1 females from crosses between isogenic tolerant/sensitive lines and Harwich males. (XLS)

S19 Table Radiation sensitive of sterile and fertile alleles and controls. (XLSX)

S20 Table Number of novel SNPs and indels in population B RIL genomes. (XLSX)

S21 Table qPCR estimates of P-element derived mRNA abundance in dysgenic ovarian RNA. (XLSX)
