# Supplementary material for: Genetic variation in P-element dysgenic sterility is associated with double-strand break repair and alternative splicing of TE transcripts
Source: PLoS Genet. 2022 Dec 7;18(12):e1010080. doi: 10.1371/journal.pgen.1010080 (PMC9762592; doi:10.1371/journal.pgen.1010080)
Supplement: S1 Fig — a) Mean expression of genes located in the pericentromere, euchromatin, telomere and the fourth chromosome from RILs carrying each of the eight B founder genotypes at the QTL-3d region. Error bars represent the standard deviation among mean expression levels of different genes. The sterile/B6 (light green) shows high pericentromeric gene expression compared to the fertile strains (dark green) (Anova; F6,494 = 7.775, P < 5.24e-08). The letters indicate significantly different expression levels based on Tukey-HSD comparisons between RILs with different founder alleles. (PDF) [file pgen.1010080.s001.pdf]

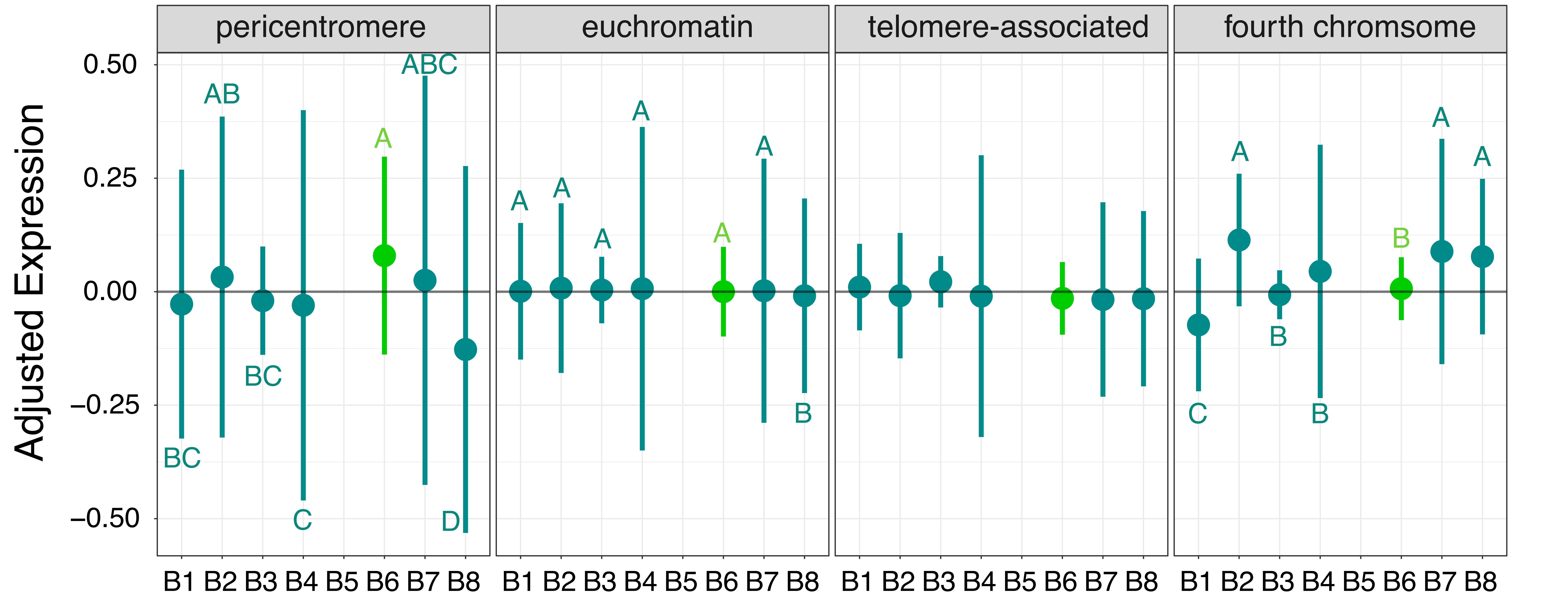
pericentromere
euchromatin
telomere-associated
fourth chromsome
Adjusted Expression
0.50
0.25
0.00
−0.25
−0.50
AB
ABC
A
BC
BC
C
D
A
A
A
A
A
A
B
A
A
A
B
B
C
B
B1 B2 B3 B4 B5 B6 B7 B8

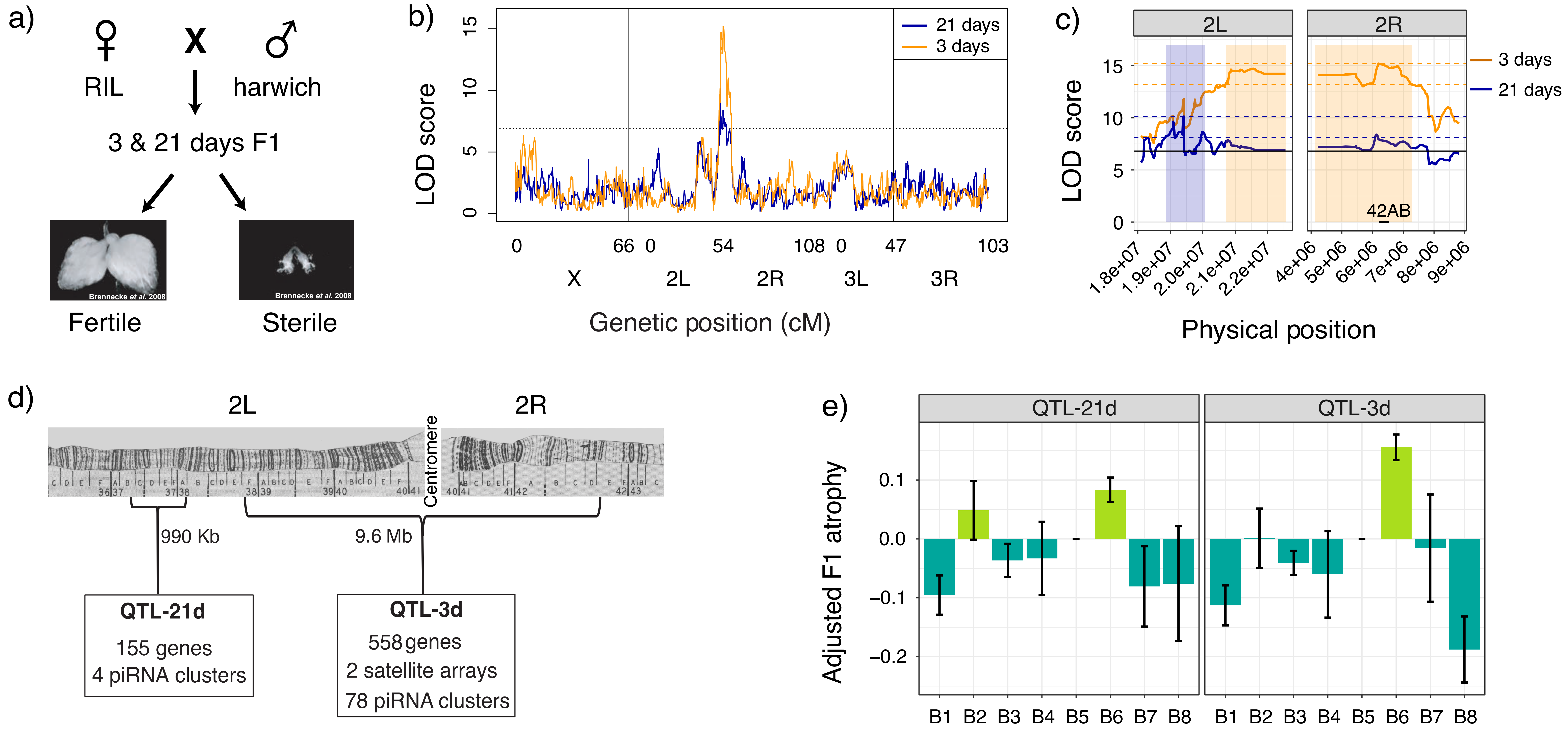
a)
♀
X
♂
RIL
harwich
3 & 21 days F1
Brennecke et al. 2008
Brennecke et al. 2008
Fertile
Sterile
b)
21 days
3 days
LOD score
0
5
10
15
0
66
0
54
108
0
47
103
X
2L
2R
3L
3R
Genetic position (cM)
c)
2L
2R
3 days
21 days
LOD score
0
5
10
15
42AB
1.8e+07
1.9e+07
2.0e+07
2.1e+07
2.2e+07
4e+06
5e+06
6e+06
7e+06
8e+06
9e+06
Physical position
d)
2L
2R
Centromere
990 Kb
9.6 Mb
QTL-21d
155 genes
4 piRNA clusters
QTL-3d
558 genes
2 satellite arrays
78 piRNA clusters
e)
QTL-21d
QTL-3d
Adjusted F1 atrophy
0.1
0.0
−0.1
−0.2
B1
B2
B3
B4
B5
B6
B7
B8
B1
B2
B3
B4
B5
B6
B7
B8

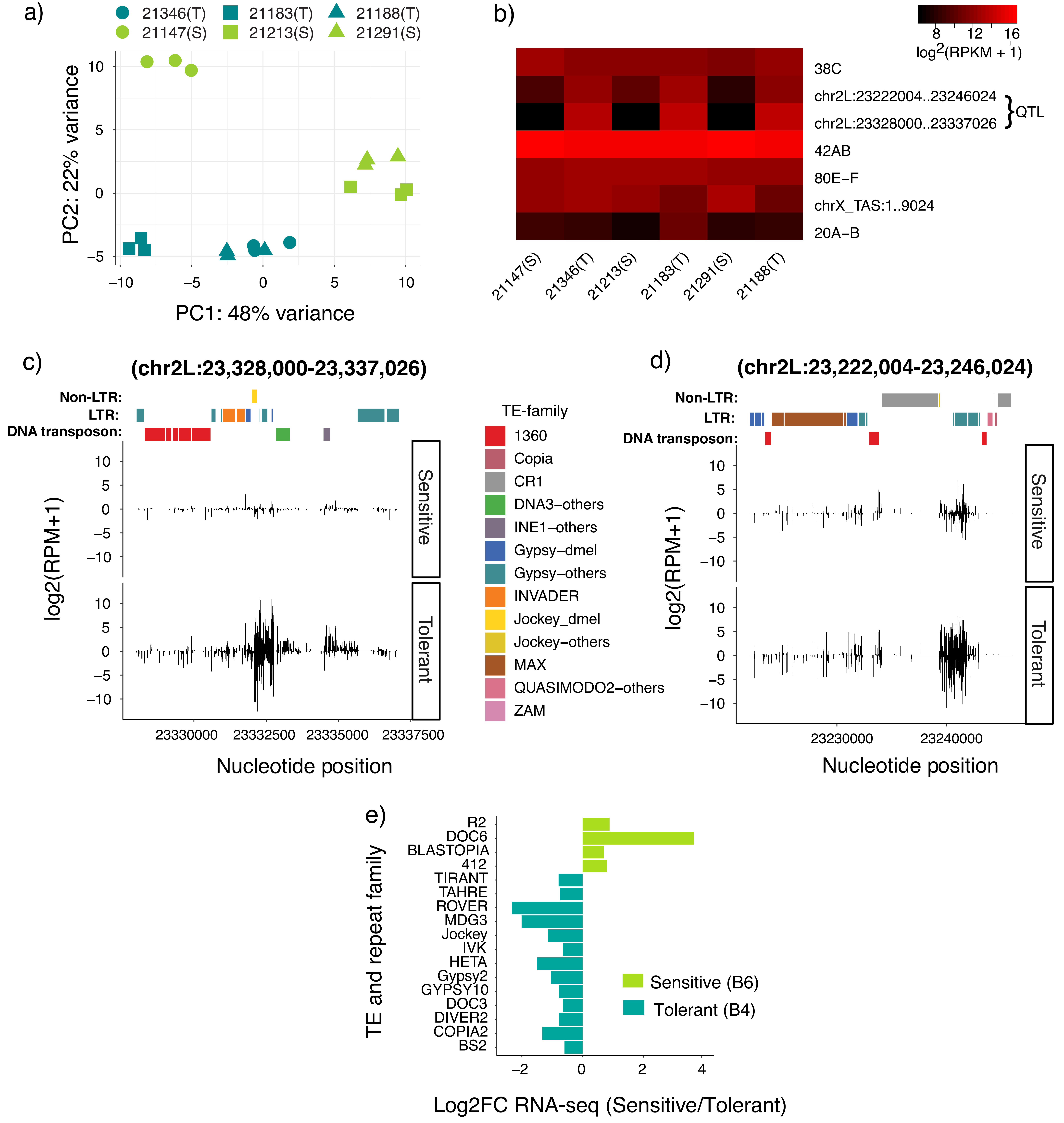

a)
21346(T)
21183(T)
21188(T)
21147(S)
21213(S)
21291(S)
PC2: 22% variance
PC1: 48% variance
b)
log2(RPKM + 1)
38C
chr2L:23222004..23246024
chr2L:23328000..23337026
QTL
42AB
80E–F
chrX_TAS:1..9024
20A–B
21147(S)
21346(T)
21213(S)
21183(T)
21291(S)
21188(T)
c)
(chr2L:23,328,000-23,337,026)
Non-LTR:
LTR:
DNA transposon:
Sensitive
Tolerant
log2(RPM+1)
23330000
23332500
23335000
23337500
Nucleotide position
TE-family
1360
Copia
CR1
DNA3–others
INE1–others
Gypsy–dmel
Gypsy–others
INVADER
Jockey_dmel
Jockey–others
MAX
QUASIMODO2–others
ZAM
d)
(chr2L:23,222,004-23,246,024)
Non-LTR:
LTR:
DNA transposon:
Sensitive
Tolerant
log2(RPM+1)
23230000
23240000
Nucleotide position
e)
TE and repeat family
R2
DOC6
BLASTOPIA
412
TIRANT
TAHRE
ROVER
MDG3
Jockey
IVK
HETA
Gypsy2
GYPSY10
DOC3
DIVER2
COPIA2
BS2
Sensitive (B6)
Tolerant (B4)
Log2FC RNA-seq (Sensitive/Tolerant)

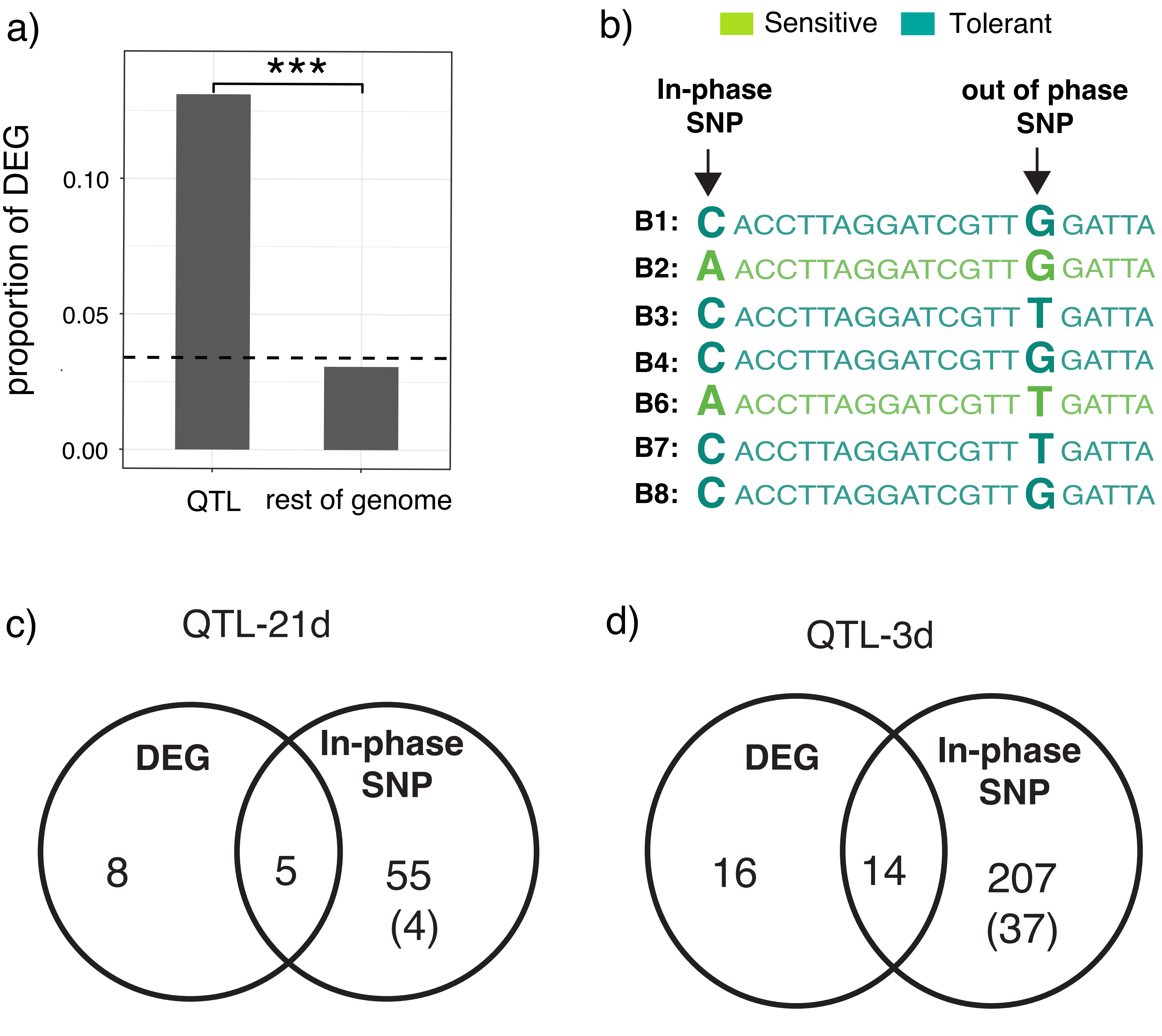
a)
***
proportion of DEG
0.10
0.05
0.00
QTL
rest of genome
b)
Sensitive
Tolerant
In-phase SNP
out of phase SNP
B1: C ACCTTAGGATCGTT G GATTA
B2: A ACCTTAGGATCGTT G GATTA
B3: C ACCTTAGGATCGTT T GATTA
B4: C ACCTTAGGATCGTT G GATTA
B6: A ACCTTAGGATCGTT T GATTA
B7: C ACCTTAGGATCGTT T GATTA
B8: C ACCTTAGGATCGTT G GATTA
c)
QTL-21d
DEG
In-phase SNP
8
5
55
(4)
d)
QTL-3d
DEG
In-phase SNP
16
14
207
(37)

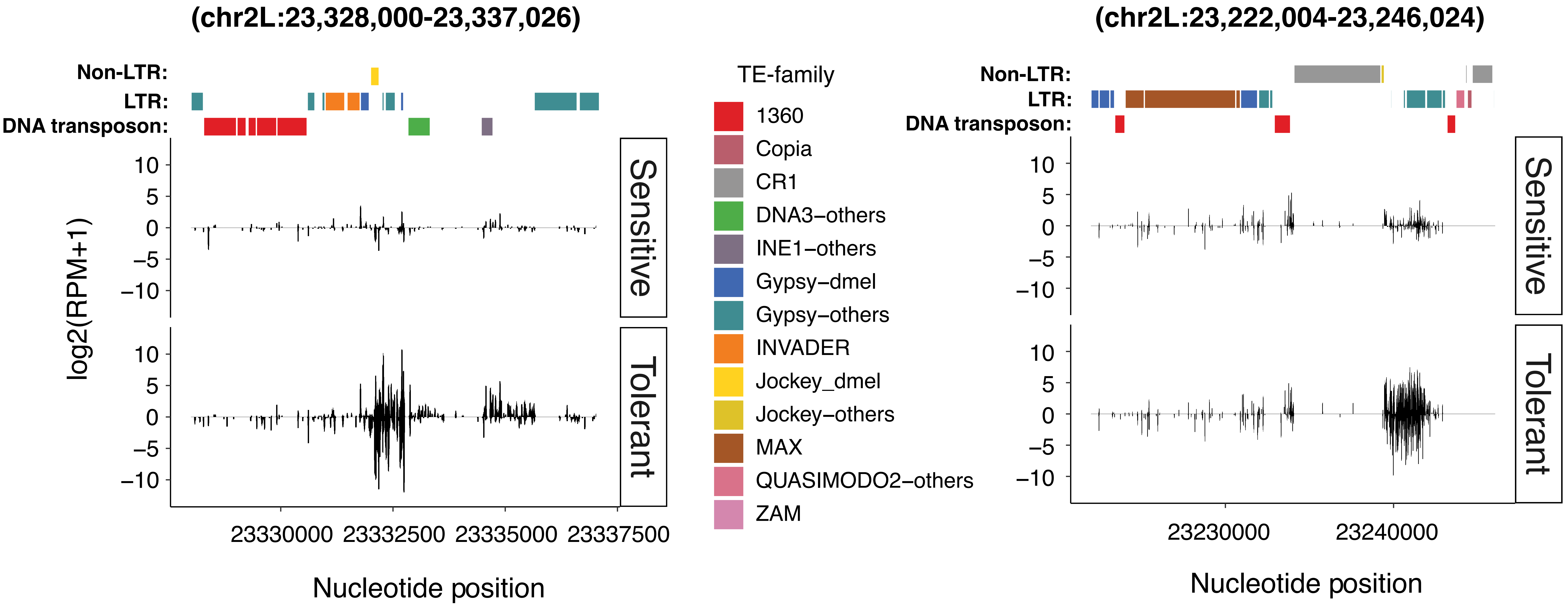

Figure S2) Expression profile of QTL piRNA clusters in sensitive and tolerant NIL pair 2S. The piRNA expression between sensitive and tolerant genotypes from 21188-21291 NIL pairs along the two QTL piRNA clusters: 2L:23,328,000-23,337,026 and 2L:23,222,004-23,246,024, respectively. Only uniquely mapping piRNAs are considered. The TE families at the top of each panel are represented by different colors. TE-others represent the repeat families coming from sibling species of D. melanogaster. Positive value indicates piRNAs mapped to the sense strand of the reference genome and negative value indicates those from the antisense strand. The piRNA cluster expression levels are estimated by log2 scale transformed of reads per million mapped reads [log2(RPM+1)].

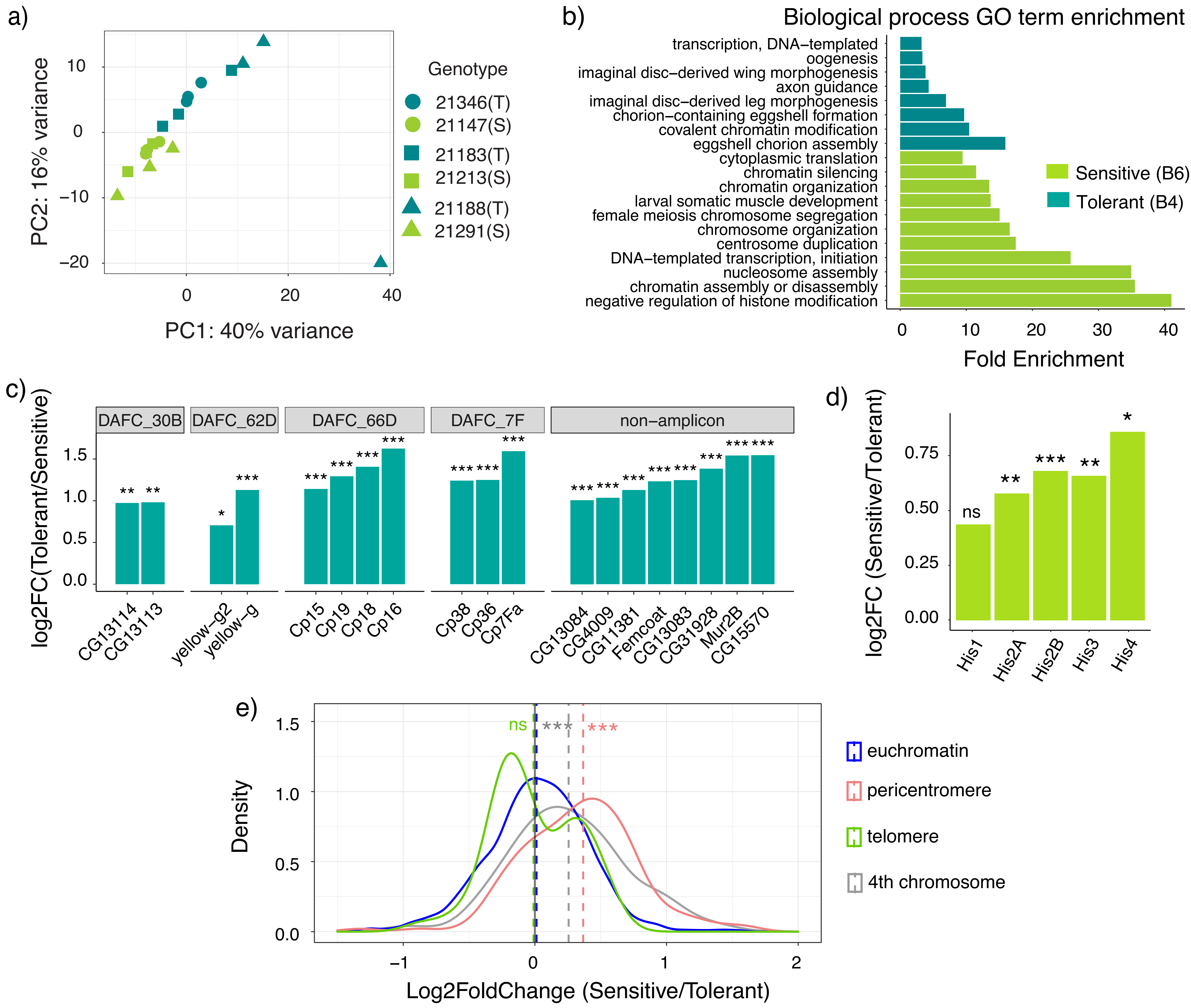
a)
Genotype
21346(T)
21147(S)
21183(T)
21213(S)
21188(T)
21291(S)
PC1: 40% variance
PC2: 16% variance
b)
Biological process GO term enrichment
transcription, DNA−templated
oogenesis
imaginal disc−derived wing morphogenesis
axon guidance
imaginal disc−derived leg morphogenesis
chorion−containing eggshell formation
covalent chromatin modification
eggshell chorion assembly
cytoplasmic translation
chromatin silencing
chromatin organization
larval somatic muscle development
female meiosis chromosome segregation
chromosome organization
centrosome duplication
DNA−templated transcription, initiation
nucleosome assembly
chromatin assembly or disassembly
negative regulation of histone modification
Sensitive (B6)
Tolerant (B4)
Fold Enrichment
c)
DAFC_30B
DAFC_62D
DAFC_66D
DAFC_7F
non−amplicon
log2FC(Tolerant/Sensitive)
CG13114
CG13113
yellow−g2
yellow−g
Cp15
Cp19
Cp18
Cp16
Cp38
Cp36
Cp7Fa
CG13084
CG4009
CG11381
Femcoat
CG13083
CG31928
Mur2B
CG15570
d)
log2FC (Sensitive/Tolerant)
ns
His1
His2A
His2B
His3
His4
e)
Density
Log2FoldChange (Sensitive/Tolerant)
ns
euchromatin
pericentromere
telomere
4th chromosome

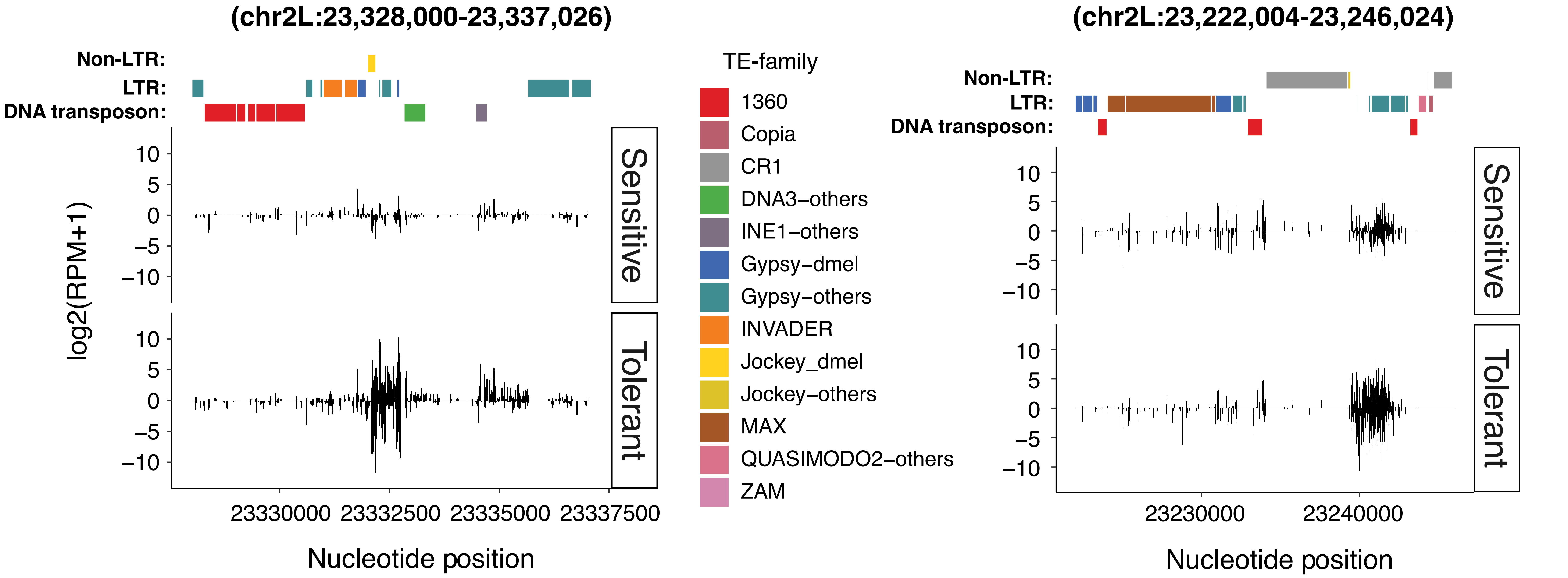

Figure S3) Expression profile of QTL piRNA clusters in sensitive and tolerant NIL pair 3 . The piRNA expression between sensitive and tolerant genotypes from 21346-21147 NIL pairs along the two QTL piRNA clusters: 2L:23,328,000-23,337,026 and 2L:23,222,004-23,246,024, respectively. Only uniquely mapping piRNAs are considered. The TE families at the top of each figure are represented by different colors. TE-others represent the repeat families coming from sibling species of D. melanogaster. Positive value indicates piRNAs mapped to the sense strand of the reference genome and negative value indicates those from the antisense strand. The piRNA cluster expression levels are estimated by log2 scale transformed of reads per million mapped reads [log2(RPM+1)].
