# Supplementary figures and images for: Genetic variation in P-element dysgenic sterility is associated with double-strand break repair and alternative splicing of TE transcripts
Source: PLoS Genet. 2022 Dec 7;18(12):e1010080. doi: 10.1371/journal.pgen.1010080 (PMC9762592; doi:10.1371/journal.pgen.1010080)

(chr2L:23,328,000-23,337,026)

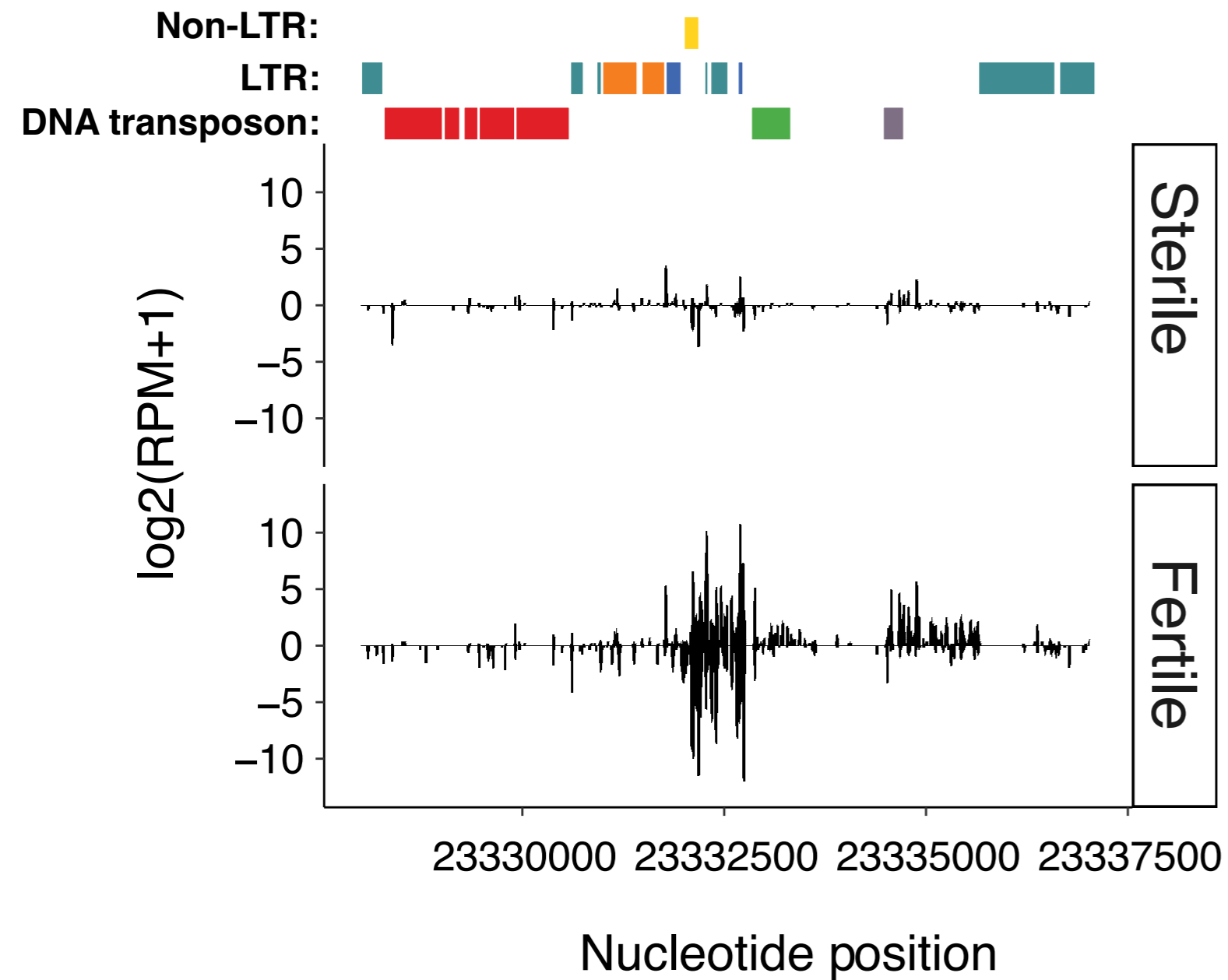

TE-family

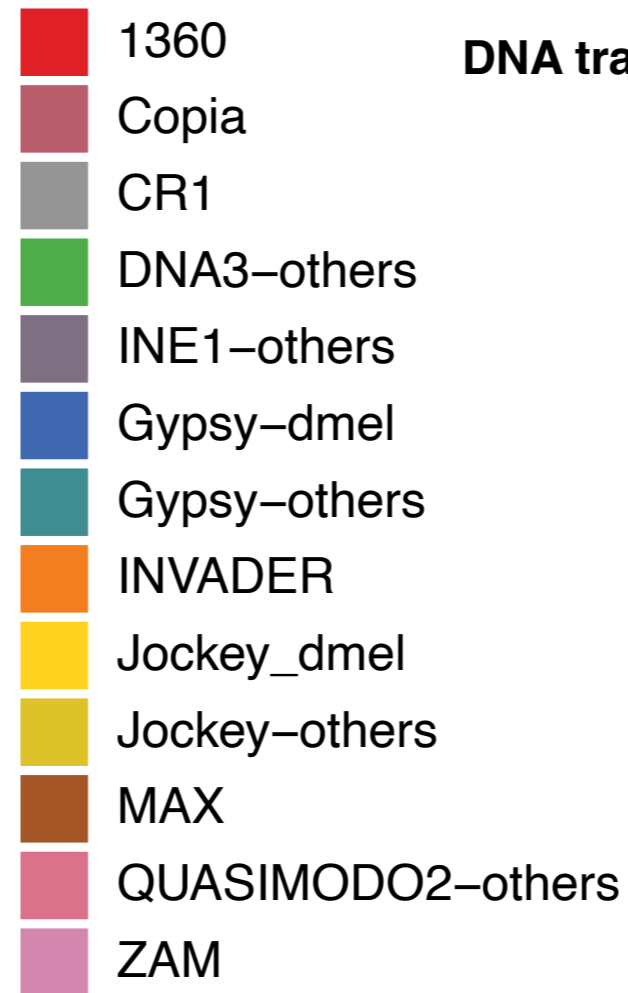

(chr2L:23,222,004-23,246,024)

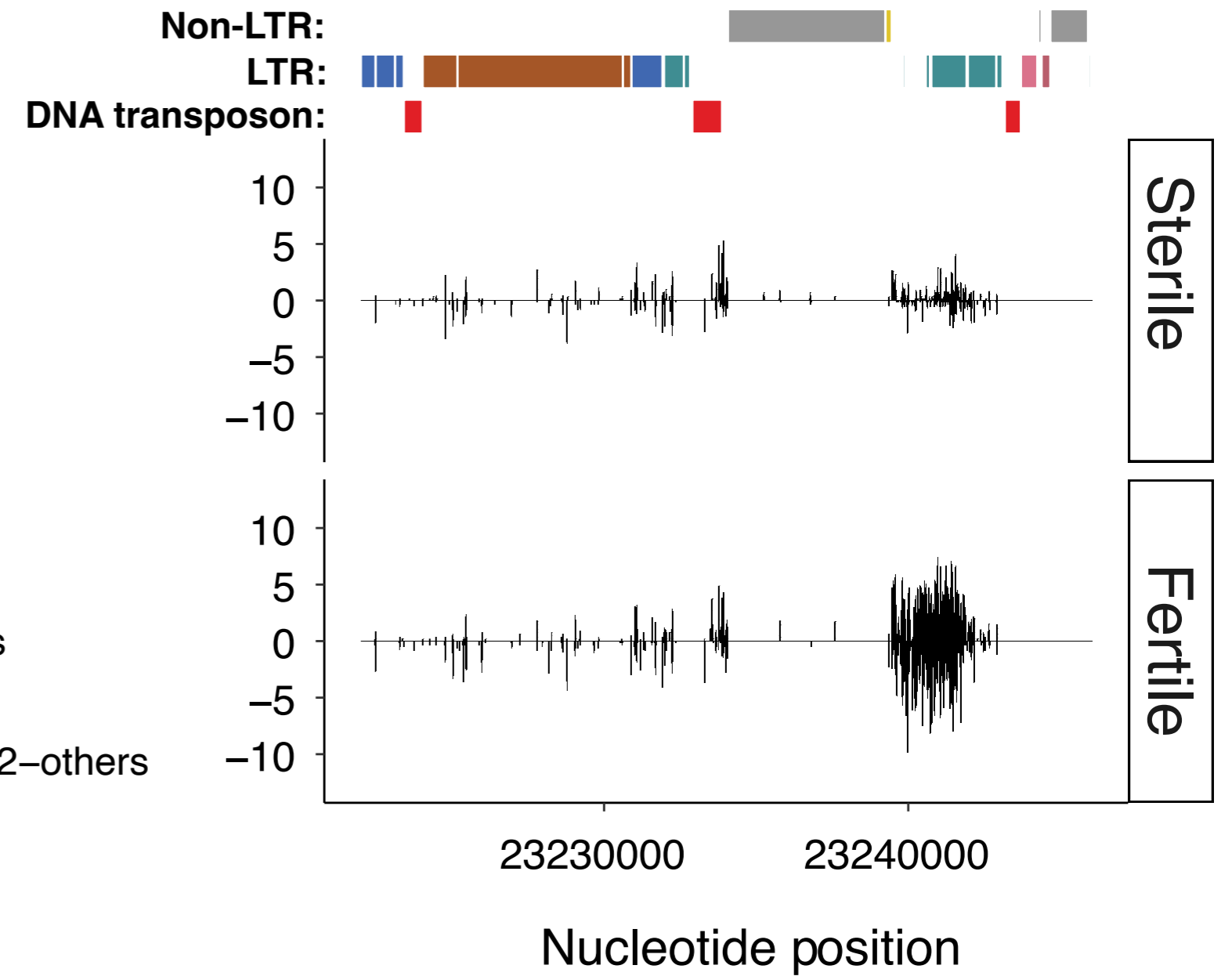

Supplement: S2 Fig — The piRNA expression between sterile and fertile genotypes from the 21188–21291 RIL pair along the two QTL piRNA clusters: 2L:23,328,000–23,337,026 and 2L:23,222,004–23,246,024, respectively. Only uniquely mapping piRNAs are considered. The TE families at the top of each panel are represented by different colors. TE-others represent the repeat families coming from sibling species of D. melanogaster. Positive value indicates piRNAs mapped to the sense strand of the reference genome and negative value indicates those from the antisense strand. The piRNA cluster expression levels are estimated by log2 scale transformed of reads per million mapped reads [log2(RPM+1)]. (PDF) [file pgen.1010080.s002.pdf]

(chr2L:23,328,000-23,337,026)

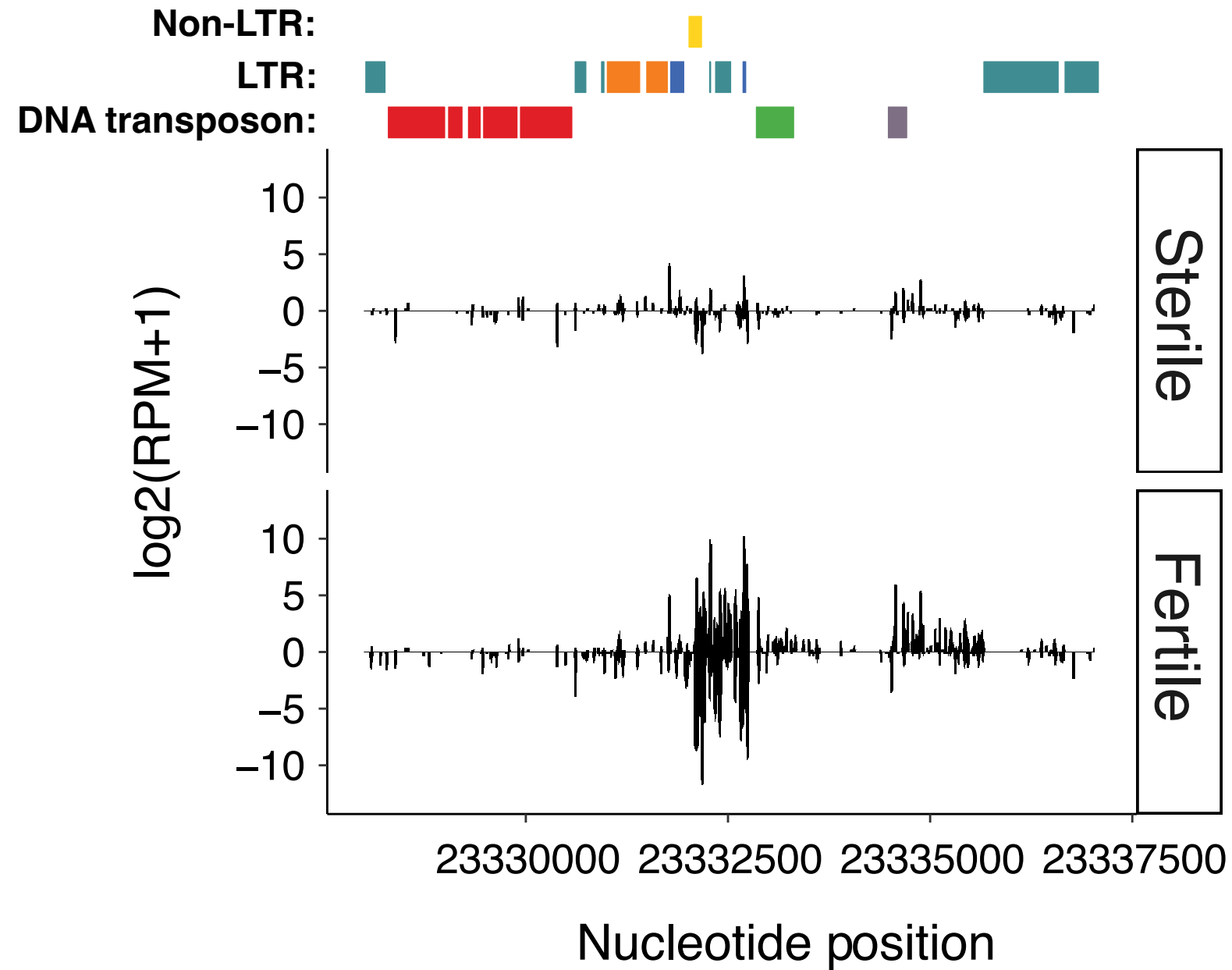

TE-family

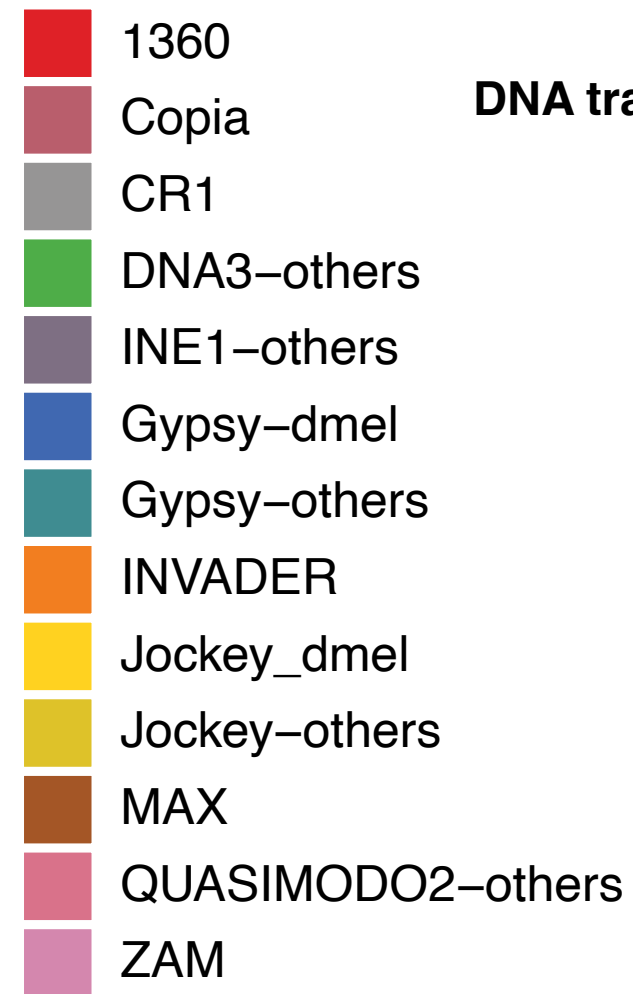

(chr2L:23,222,004-23,246,024)

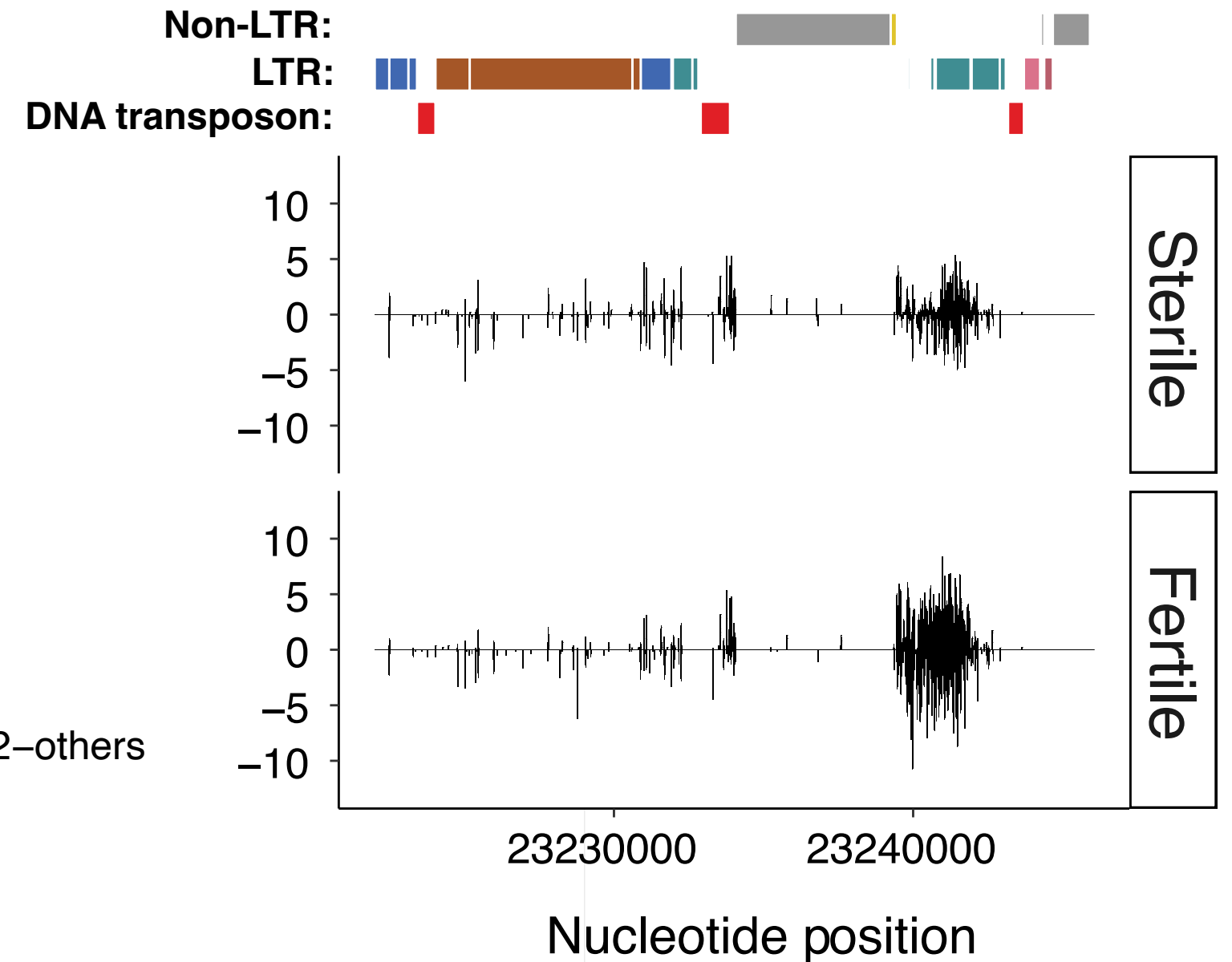

Supplement: S3 Fig — The piRNA expression between sterile and fertile genotypes from the 21346–21147 RIL pair along the two QTL piRNA clusters: 2L:23,328,000–23,337,026 and 2L:23,222,004–23,246,024, respectively. Only uniquely mapping piRNAs are considered. The TE families at the top of each Fig are represented by different colors. TE-others represent the repeat families coming from sibling species of D. melanogaster. Positive value indicates piRNAs mapped to the sense strand of the reference genome and negative value indicates those from the antisense strand. The piRNA cluster expression levels are estimated by log2 scale transformed of reads per million mapped reads [log2(RPM+1)]. (PDF) [file pgen.1010080.s003.pdf]

7 rounds of backcrossing

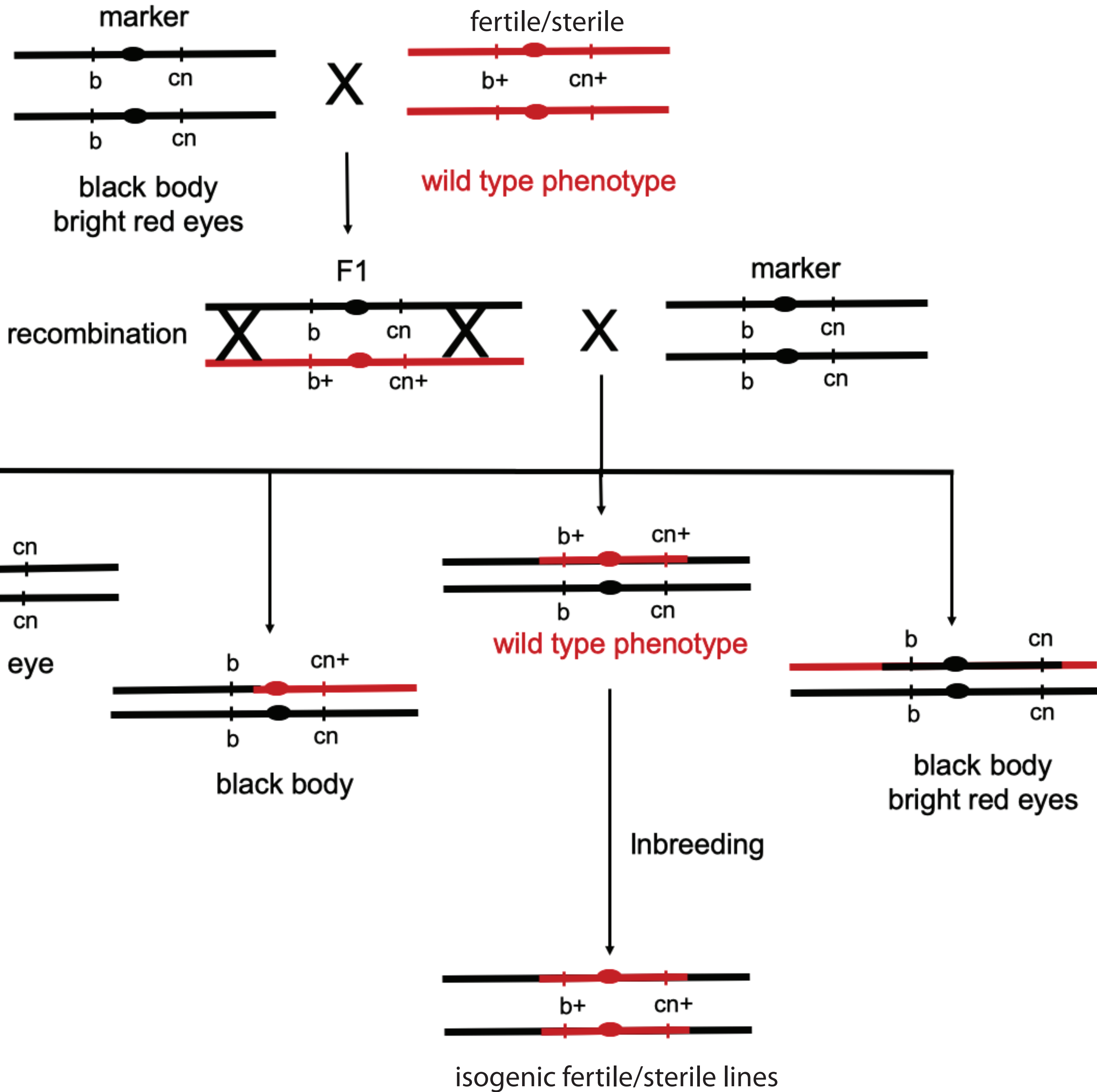

Supplement: S5 Fig — (PDF) [file pgen.1010080.s005.pdf]

log2 fold-change fertile/sterile

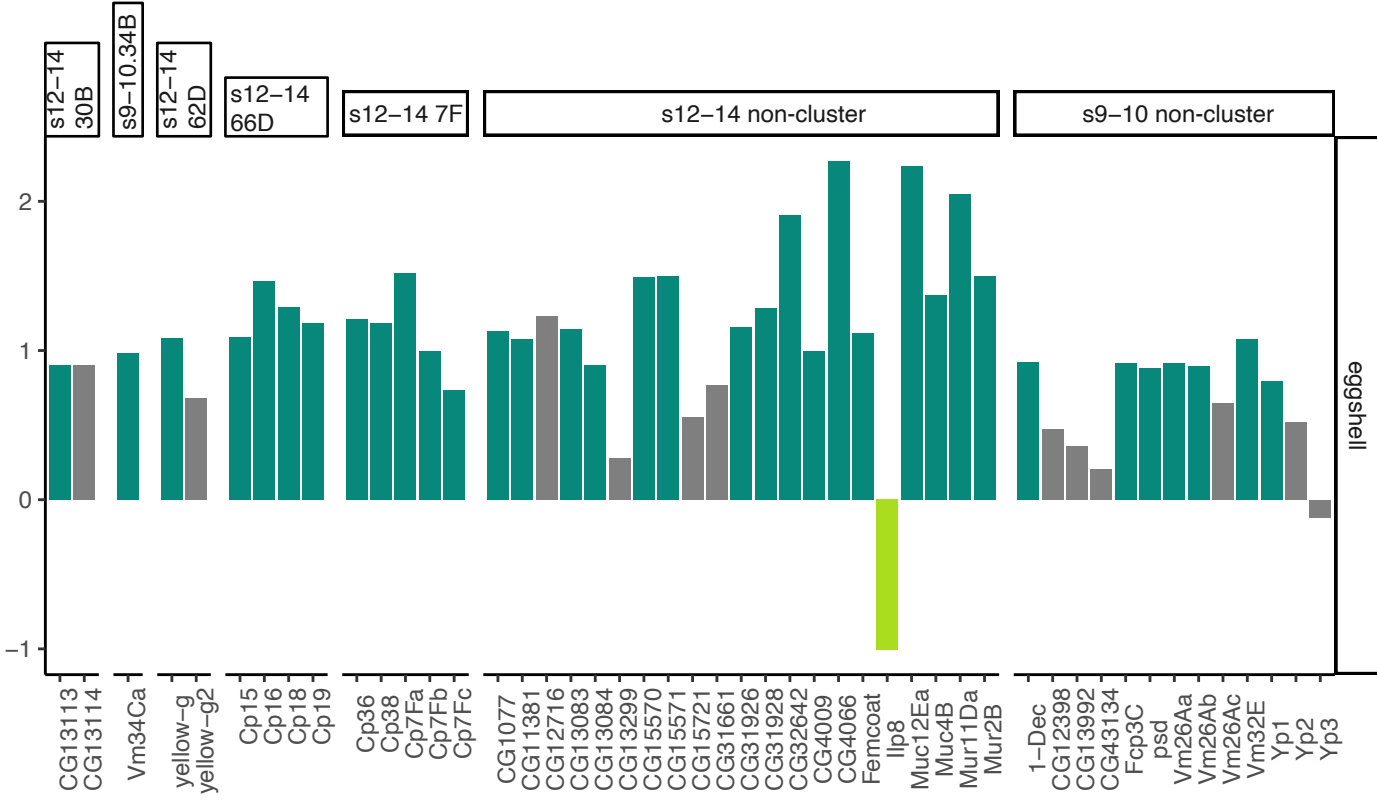

log2 fold-change fertile/sterile

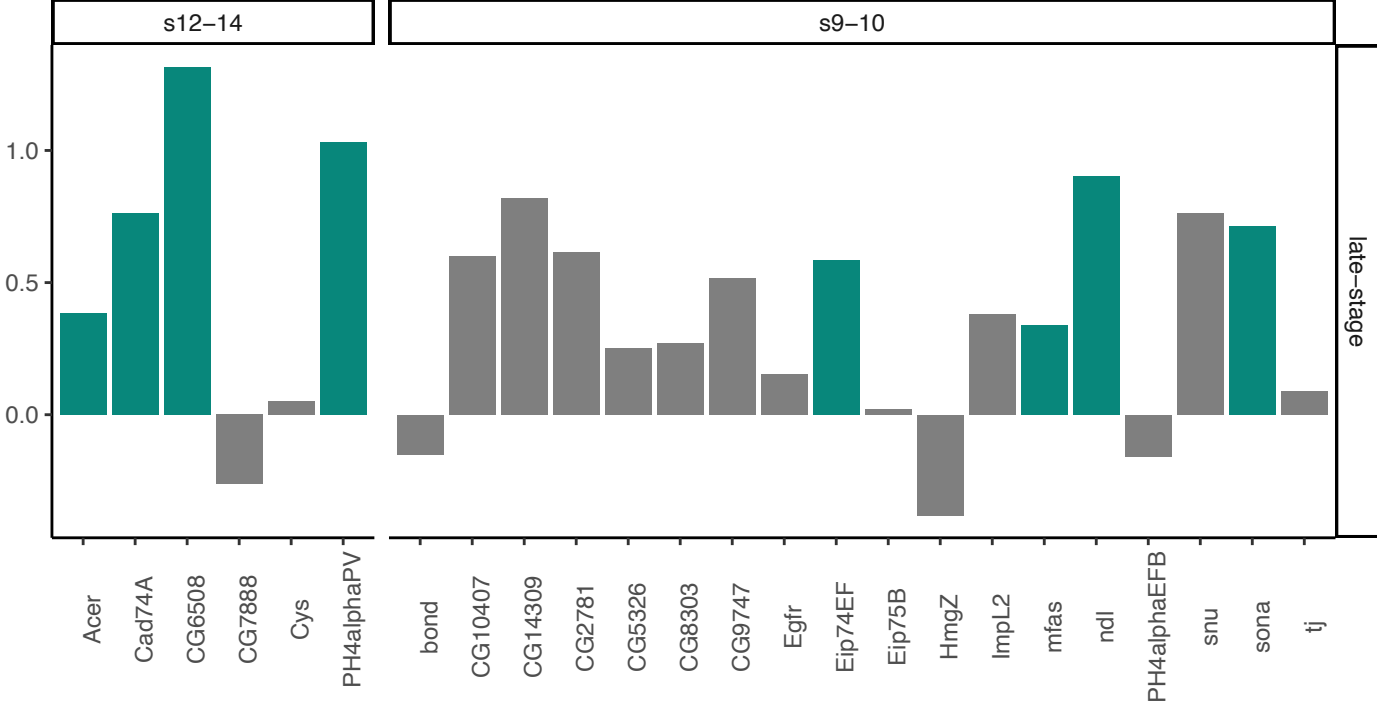

Supplement: S6 Fig — Upregulation in stage 9–10 and stage 12–14 egg chambers is from Tootle et al. [30]. Genes are separated into eggshell components (top) and non-eggshell components (bottom). Dark green bars indicate genes significantly upregulated in fertile genotypes whereas light green indicates genes upregulated in sterile genotypes. (PDF) [file pgen.1010080.s006.pdf]
