# Supplementary material for: Genetic variation in P-element dysgenic sterility is associated with double-strand break repair and alternative splicing of TE transcripts
Source: PLoS Genet. 2022 Dec 7;18(12):e1010080. doi: 10.1371/journal.pgen.1010080 (PMC9762592; doi:10.1371/journal.pgen.1010080)
Supplement: S4 Fig — Four haplotypes are compared, which comprise all possible combinations of sterility alleles at 2 QTL. The allele at the 3 day QTL is indicated first and is represented by the color of the violin plot (light green = sterile, dark green = fertile). The allele at the 21 day QTL is indicated second and represented by the color of the points on the scatter plot. Y-axis is residual variation in F1 atrophy after accounting for student experimenter and block. Among 3 day old females, haplotypes containing different alleles for the 3 day old QTL are significantly different from each other (Tukey HSD P = 0.016–0). However, haplotypes containing alternative QTL for the 21d only do not differ from each other (Tukey HSD P>0.74). This suggests phenotypic variation in 3 day old females is not influenced by their genotype at the 21 day QTL. In contrast, among 21 day old females tolerant alleles in both QTL loci are required to significantly decrease sterility below the sterile allele containing haplotypes (Tukey HSD P = 0.01–0). (PDF) [file pgen.1010080.s004.pdf]

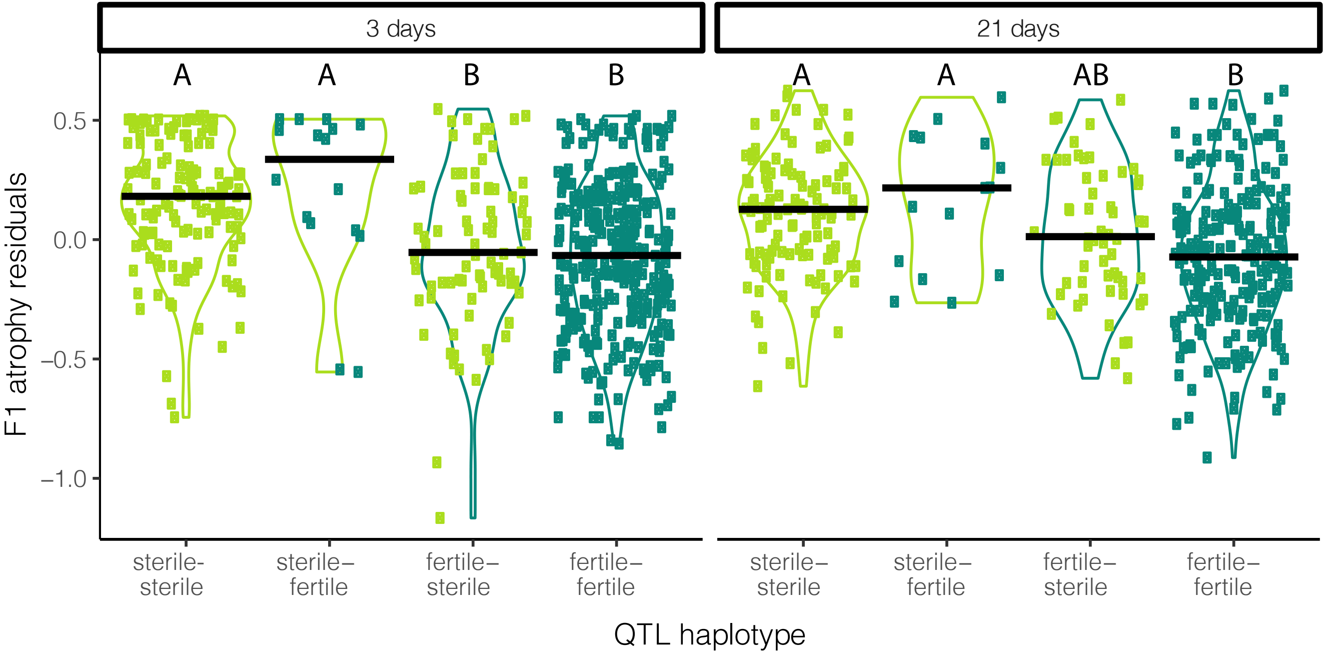

3 days
21 days
A
A
B
B
A
A
AB
B
0.5
0.0
−0.5
−1.0
F1 atrophy residuals
sterile-sterile
sterile–fertile
fertile–sterile
fertile–fertile
sterile–sterile
sterile–fertile
fertile–sterile
fertile–fertile
QTL haplotype
